# Microbial dysbiosis in cholangiocarcinoma

**DOI:** 10.3389/fmicb.2026.1727736

**Published:** 2026-01-27

**Authors:** Yunjia Liu, Shaohui Huang, Yang Zhang, Yuankun Zhang, Yunfei Xu, Yongchang Tang, Sen Guo, Zongli Zhang

**Affiliations:** Department of General Surgery, Qilu Hospital, Shandong University, Jinan, Shandong, China

**Keywords:** bile microbiome, cholangiocarcinoma, gut microbiome, intratumoral microbiome, microbial dysbiosis

## Abstract

Cholangiocarcinoma (CCA) is a highly aggressive malignancy of the biliary epithelium, with its incidence and mortality rates continuing to rise worldwide. Advances in high-throughput sequencing and metabolomic technologies have intensified interest in elucidating the role of the microbiome in CCA. Microbial dysbiosis may contribute to tumor initiation and progression by inducing chronic inflammation, altering metabolic pathways, and modulating the immune microenvironment. Moreover, these microbial alterations have been associated with therapeutic resistance, underscoring their potential impact on disease progression and treatment outcomes. This review summarizes the potential origins of intratumoral microorganisms and the microbiome alterations associated with distinct CCA subtypes. Crucially, we critically evaluate the methodological challenges inherent to low-biomass biliary samples—including contamination risks and confounding factors such as cholestasis and medical interventions—and distinguish between associative and causal evidence in current literature. Collectively, this work aims to provide a rigorous theoretical framework and novel insights for microbiome-based strategies in the early diagnosis and treatment of CCA.

## 1 Introduction

CCA is a heterogeneous malignancy arising from the biliary tract and is anatomically classified into three subtypes: intrahepatic CCA (iCCA), perihilar CCA (pCCA), and distal CCA (dCCA). These subtypes exhibit distinct biological and clinical characteristics, leading to marked differences in surgical management and patient prognosis (Harrison and Visser, 2024; Tang et al., 2024; Huang et al., 2025). Despite advances in diagnostic and therapeutic strategies, CCA remains a highly lethal malignancy. Owing to its insidious onset, aggressive biological behavior, and high recurrence rate after surgical resection, the 5-year survival rate for patients with advanced disease remains stagnant at approximately 5%, resulting in an extremely poor overall prognosis (Valle et al., 2017; Khan and Dageforde, 2019; Li et al., 2024).

In recent years, accumulating evidence has suggested that microbial exposure may play a potential role in the pathogenesis of CCA (Rao et al., 2021; Ye et al., 2023; Park, 2024). Various parts of the human body, including the bile duct, harbor diverse microbial communities that, together with their associated molecular products, constitute the microbiome (Aggarwal et al., 2023; Xiao et al., 2024; Armengaud, 2025). The vast genetic diversity of these microorganisms underpins their remarkable metabolic capacity and plays a pivotal role in regulating host tissue specificity and immune function (Sender et al., 2016; Koneru et al., 2023; Xiao et al., 2024; Armengaud, 2025). Lifestyle, diet, disease status, infections, and antibiotic use can markedly influence the relative abundance and diversity of the microbiome (Yatsunenko et al., 2012; Xiao et al., 2024). The intestinal microbiome plays a critical role in maintaining mucosal homeostasis, epithelial barrier integrity, energy metabolism, pathogen resistance, and immune stability, and also shapes host–microbe interactions (Bäckhed et al., 2005; Heintz-Buschart and Wilmes, 2018; Takeuchi et al., 2024). Dysbiosis, or disruption of this balance, has been implicated in the development of various diseases, including cancer (Altveş et al., 2020; Sepich-Poore et al., 2021). Reported associations include gastric cancer (Stewart et al., 2020; Liu et al., 2024; Zeng et al., 2024), colorectal cancer (Clay et al., 2022; Dougherty and Jobin, 2023; Wang et al., 2023), liver cancer (Schöler and Schnabl, 2024; Ke et al., 2025), CCA (Rao et al., 2021; Chai et al., 2023; Ye et al., 2023) among others. The advent of next-generation sequencing (NGS) technology has greatly facilitated microbiome research, sparking a surge of studies on the interactions between the human microbiome and cancer (Bhatt et al., 2017; Davidson and Epperson, 2018; Ye et al., 2019; Wang et al., 2025). Increasing evidence has revealed a close association between the microbiome and CCA.

To ensure a comprehensive and reproducible synthesis of current evidence, we conducted a structured literature search across PubMed, Web of Science, and Embase up to November 2025. Search terms combined disease-related keywords (“cholangiocarcinoma,” “CCA,” “biliary tract cancer”) with microbiome-related terms (“microbiome,” “microbiota,” “dysbiosis,” “fungi,” “virome,” “bacteria”) and mechanistic contexts (“metabolomics,” “tumor microenvironment,” “immunotherapy”). Inclusion criteria encompassed original research and systematic reviews published in English that characterized microbiome composition (e.g., 16S rRNA sequencing, shotgun metagenomics) or explored functional mechanisms in human CCA or relevant animal models. Conference abstracts, commentaries, and studies focusing solely on viral hepatitis–related carcinogenesis without microbial analysis were excluded. As this work is a narrative review, formal PRISMA-style scoring was not applied. Instead, we developed a customized Evidence Grading Framework, categorizing microbiome–CCA findings from observational association to C4 mechanistic validation to strengthen transparency and guide interpretation of evidence quality.

Accordingly, this review aims to move beyond a mere summary of reported associations to provide a critical and comprehensive analysis of the intricate relationship between the microbiome and CCA. We systematically examine the potential roles of the microbiome in the initiation, progression, and treatment of this malignancy, with a specific focus on: (1) dissecting microbial alterations across distinct CCA subtypes; (2) critically evaluating the strength of evidence distinguishing causality from correlation, while addressing key methodological challenges and confounding factors; (3) elucidating the mechanistic pathways driving tumorigenesis, including emerging non-bacterial components like fungi and viruses; and (4) proposing standardized frameworks for future research to accelerate clinical translation.

## 2 Microbial alterations and potential origins in CCA

Accumulating evidence indicates that patients with CCA exhibit widespread microbial alterations across multiple body sites, including the gut, oral cavity, bile, blood, and tumor tissues (Jia et al., 2020; Lee et al., 2020; Li et al., 2022; Rao et al., 2022; Xin et al., 2024). These findings highlight that CCA-associated microbial dysbiosis is a systemic and multi-compartmental phenomenon. Under physiological conditions, the biliary tract is generally considered sterile owing to the continuous flow of bile and the intrinsic antimicrobial activity of bile acids. However, in pathological states such as biliary obstruction, chronic inflammation, or malignant transformation, this sterility barrier can be compromised, allowing microbial invasion, colonization, and persistence within the biliary system (Verdier et al., 2015; Han et al., 2021). The disruption of this barrier and the detection of microorganisms within tumor tissues underscore the need to elucidate the potential origins and dissemination routes of intratumoral microbiota in CCA.

Multiple studies have demonstrated the presence of intratumoral microorganisms in CCA; however, definitive conclusions regarding their origins remain elusive (Nejman et al., 2020; Ma et al., 2024). Considering the existence of the gut–liver axis and the biliary–enteric circulation, interactions between intestinal microbes and intratumoral bacteria may occur, suggesting that the intratumoral microbiota in CCA could, at least in part, originate from gut microorganisms (Chiang and Ferrell, 2018; Liu and Zhang, 2022; Chai et al., 2023). The portal venous system represents a crucial route for the translocation of gut-derived microbes into the liver and biliary tree. For example, *Escherichia coli* carrying the virulence regulator VirF can disrupt the gut vascular barrier, enabling its translocation to the liver through the portal vein, where it contributes to the formation of a premetastatic niche in colorectal cancer (Bertocchi et al., 2021). Moreover, patients with primary sclerosing cholangitis (PSC, an independent risk factor for CCA) and CCA often exhibit impaired intestinal barrier function, which increases gut permeability and facilitates the translocation of gut microbes and microbial metabolites into the hepatobiliary system (Rao et al., 2021; Hov and Karlsen, 2023). Several studies have also demonstrated that, in patients with gallstone disease, the biliary and duodenal microbiota exhibit remarkably similar diversity and compositional profiles. This similarity provides indirect evidence that duodenal or intestinal microbes might serve as a potential source of biliary microbiota (Ye et al., 2016; Han et al., 2021). Although retrograde translocation of intestinal bacteria remains a plausible contributor to the intratumoral microbiome in CCA, additional experimental evidence is needed to confirm this hypothesis. Wang *et al.* reported a substantial overlap in bacterial genera such as *Enterococcus* and *Staphylococcus* between bile and tumor tissues in CCA patients, further supporting the notion that intratumoral bacteria may partially derive from biliary microbiota (Wang et al., 2025). Additionally, microbial communities in tumors and adjacent non-tumor tissues often display compositional similarities across cancer types, suggesting that microbial infiltration from neighboring tissues may also occur (Nejman et al., 2020). An observational study specifically demonstrated that CCA tumors share certain microbial taxa with adjacent normal tissues, providing further evidence that some intratumoral microorganisms might originate from surrounding hepatic tissue (Xin et al., 2024).

**Figure 1:**
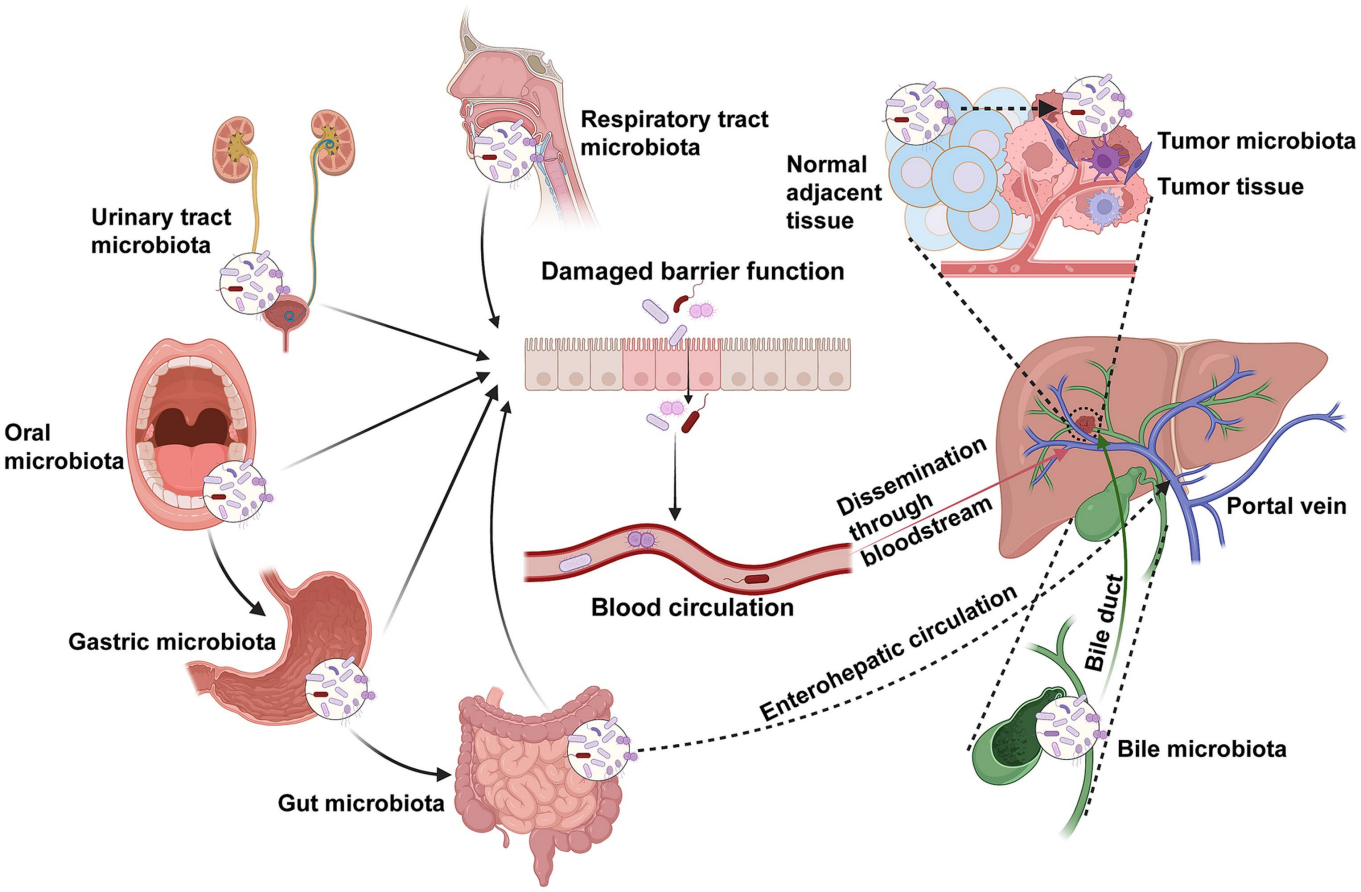
The schematic illustrates multiple potential sources and migration pathways of tumor-associated microbiota. Microorganisms may originate from various mucosal sites, including the oral cavity, respiratory tract, stomach, intestine, and urinary tract. Under pathological conditions such as barrier dysfunction or increased permeability, these microbes can translocate into the bloodstream and disseminate to distant organs. The gut–liver axis and biliary–enteric circulation provide additional routes for microbial migration, enabling bacteria from the intestine or bile to reach the liver and tumor tissues through the portal venous system. Moreover, microorganisms from adjacent normal tissues may also infiltrate the tumor microenvironment. Collectively, these routes contribute to the complex and multifactorial origins of the intratumoral microbiota in cholangiocarcinoma.

Microorganisms from other mucosal sites outside the intestine—including the oral cavity, stomach, upper respiratory tract, and genitourinary tract—can also access tumor tissues through the bloodstream (Abed et al., 2016; Sun et al., 2020; Ma et al., 2024; Ma et al., 2024; Yu et al., 2024). These mucosal surfaces are continuously exposed to the external environment and harbor diverse microbial communities. When mucosal barriers are compromised, microbes may enter the circulation and migrate to tumor sites, potentially guided by chemotactic gradients generated by necrotic tumor debris (Helmink et al., 2019; Ma et al., 2024). Red blood cells may facilitate microbial translocation and immune evasion by serving as vehicles for bacterial concealment (Qi et al., 2020). Furthermore, the rich vascularization and unique microenvironment of tumor tissues provide favorable conditions for microbial colonization and survival (Ke et al., 2025; Figure 1).

Collectively, these pathways underscore the complexity and diversity of the microbial origins within the biliary system. Elucidating the origins and dissemination routes of tumor-associated microbiota is essential for understanding their biological roles in shaping the tumor microenvironment, modulating immune responses, and potentially influencing therapeutic efficacy. Although current evidence provides valuable insights into the possible sources and transmission mechanisms of microorganisms in CCA, further comprehensive and mechanistic investigations are required to validate these hypotheses and clarify the causal links between microbial colonization and tumor progression. However, the detection of these translocated microbes and the interpretation of their origins are heavily influenced by the unique physiological constraints of the biliary tract (such as low biomass) and the complex clinical management of CCA patients. Therefore, prior to detailing the specific microbial dysbiosis across different niches, it is imperative to critically evaluate the methodological hurdles and confounding variables that frame current research.

## 3 Potential confounders and methodological challenges in CCA microbiome research

The rapid advancement of CCA microbiome research is tempered by complex clinical confounders and specific methodological challenges. A critical understanding of these limitations is fundamental for accurately interpreting and comparing findings across studies.

### 3.1 Potential confounders influence the microbial community in CCA

The interpretation of microbiome alterations in CCA is complicated by numerous clinical and procedural confounders that substantially shape microbial communities across different anatomical sites. First, biliary obstruction is common in CCA and can cause stasis, local hypoxia, and increased bacterial translocation, thereby altering both bile and gut microbiota. Cholangitis, often secondary to obstruction, induces acute inflammation and promotes overgrowth of enteric organisms in the biliary tree (Vieira-Silva et al., 2019; Qian et al., 2025). Second, endoscopic procedures—particularly ERCP and biliary stent placement—directly introduce exogenous microbes from the duodenum, disrupt the native biliary environment, and create biofilm-rich niches on stent surfaces, profoundly modifying bile microbial profiles (Al-Kabban et al., 2025; Kayashima et al., 2025). Third, antibiotics administered for cholangitis or peri-procedural prophylaxis can dramatically reshape both gut and bile microbiota, reduce microbial diversity, and selectively enrich resistant taxa, complicating comparisons across patients and studies (Patangia et al., 2022; Sitthirak et al., 2022). Diet, which influences gut microbial composition and bile acid metabolism, represents an additional unmeasured source of inter-study heterogeneity (Xiao et al., 2024).

Underlying liver disease also acts as a major confounder. Cirrhosis is associated with altered intestinal permeability, bacterial translocation, and characteristic dysbiosis, while PSC—a strong predisposing condition for CCA—has its own distinct biliary and gut microbial signature that may precede or mimic tumor-related changes (Miyabe et al., 2022).

Parasite exposure (e.g., *Opisthorchis viverrini*, *Clonorchis sinensis*), relevant in endemic regions, can cause chronic inflammation and unique biliary microbial shifts independent of malignancy (Chng et al., 2016; Chen et al., 2023). Finally, tumor stage may influence microbial composition through differences in biliary obstruction severity, necrosis, immune changes, and metabolic alterations (Wade and Hall, 2019; Table 1).

**Table 1:** Clinical variables and confounding factors influencing CCA microbiome studies.

Confounding factor	Ecological niche	Mechanism of impact	Challenge to research	Author year
Biliary Obstruction/ Cholangitis	Bile/Intestine	Obstruction leads to reduced bile flow, stasis, and ascending bacterial colonization. This significantly increases the load of *Clostridium* in the bile and *Enterococcus* in the intestine.	Difficult to distinguish between dysbiosis caused by cholangitis and dysbiosis caused by the tumor itself.	Vieira-Silva et al. (2019) and Qian et al. (2025)
ERCP/ Stent Placement	Bile/ Tissue	Contamination introduced during ERCP (oral cavity, gastrointestinal tract) is a major source of bias in bile and biliary tissue microbiome studie. Stents act as foreign bodies, promoting biofilm formation and the enrichment of specific pathogens.	Reported high-abundance flora might be indicative of stent-related infection rather than carcinogen-driven flora.	Al-Kabban et al. (2025) and Kayashima et al. (2025)
Antibiotic/ Drug Exposure/ Diet	All	This is the most powerful confounder, rapidly reducing microbial diversity, altering community structure, and affecting tumor- intrinsic microbe-mediated drug metabolism	Patients who received antibiotic treatment shortly before sample collection must be excluded, otherwise the disease-associated microbial signature cannot be accurately reflected.	Patangia et al. (2022), Sitthirak et al. (2022), and Xiao et al. (2024)
PSC	Bile	CCA precursor that selectively enriches Fusobacterium through chronic inflammation	PSC-associated CCA must be analyzed separately from sporadic CCA, as their etiology and microbial drivers differ	Miyabe et al. (2022)
Parasitic Exposure	Tissue/ Bile	*Opisthorchis viverrini* infection in Southeast Asia drives chronic inflammation that enriches OVa-CCA-specific flora (e.g., *Bifidobacteriaceae*). *Clonorchis sinensis* infection induced *Proteobacteria* increased	When comparing different geographical or etiological subtypes, a subtype-specific healthy control or peritumoral control must be used as the benchmark.	Chng et al. (2016) and Chen et al. (2023)
Tumor Stage/ Cachexia	Intestine/ Blood	Advanced tumor stage or cachexia may indirectly affect the intestinal flora by changing nutritional status and systemic inflammatory responses, thereby confounding causal association.	Cross-sectional studies struggle to distinguish whether dysbiosis causes disease progression or disease progression causes dysbiosis (reverse causality)	Wade and Hall (2019)

Together, these factors significantly limit the comparability of microbiome findings across cohorts. Many published studies do not consistently report or adjust for these variables, leading to substantial heterogeneity and potentially confounding the attribution of specific microbial signatures to CCA itself. Standardized reporting of clinical variables, careful matching or adjustment for key confounders, and prospective study designs will be essential to improve reproducibility and causal interpretation in future CCA microbiome research.

### 3.2 Contamination control and best practices for low-biomass microbiome studies in CCA

Bile, biliary tissue, and tumor specimens represent low-biomass environments, making them particularly vulnerable to contamination from extraction reagents, sequencing kits, environmental exposure, and endoscopic or surgical procedures. Several seminal studies have demonstrated that reagent-derived microbial DNA—such as *Ralstonia*, *Sphingomonas*, *Acinetobacter*, and *Pseudomonas*—can dominate low-biomass profiles and lead to false positives if not adequately controll. Similarly, sequencing artifacts such as index hopping or barcode cross-talk can introduce spurious taxa, particularly when microbial loads are extremely low (Salter et al., 2014; Eisenhofer et al., 2019; Selway et al., 2020).

Best practices therefore require comprehensive contamination mitigation approaches. Negative controls (including extraction blanks, PCR blanks, and environmental swabs) should be processed alongside biological samples to identify background contaminants (Bokulich et al., 2020; Fierer et al., 2025). Computational filtering strategies—such as frequency-based (e.g., Decontam’s “frequency” method), prevalence-based, or statistical reagent-background subtraction—are strongly recommended to distinguish true low-abundance taxa from contaminants. Furthermore, low-biomass samples benefit from quantification of microbial DNA (e.g., qPCR of 16S rRNA genes) to assess biomass and interpret downstream profiles (Dohlman et al., 2021; Fierer et al., 2025; Griffard-Smith et al., 2025).

Against this methodological background, we systematically assessed contamination-control practices in key low-biomass microbiome studies cited in this review. Notably, many early or small-cohort studies did not report the inclusion of negative controls, the application of decontamination algorithms (e.g., Decontam), or microbial biomass quantification by qPCR. Accordingly, these studies are classified as exploratory and hypothesis-generating, and their findings should be interpreted with appropriate caution. In contrast, several tissue-based studies with larger cohorts and spatial or functional validation provide higher-level mechanistic evidence.

In CCA research, endoscopic procedures (e.g., ERCP, stent placement) and instrumental contamination can introduce exogenous microbes into bile samples, while surgical handling may introduce skin or environmental bacteria into tumor tissues. These procedural factors must be documented and controlled, as they significantly complicate cross-study comparisons (Al-Kabban et al., 2025). Differences in laboratory workflows, the absence of negative controls, and variable contamination-filtering pipelines likely contribute to inconsistent reports of “bile microbiota” or “intratumoral microbiota” across existing studies (Agudelo and Miller, 2025).

To improve reproducibility, future bile and tissue microbiome studies should adopt standardized workflows with strict contamination monitoring, rigorous inclusion of negative controls, transparent reporting of biomass, and use of validated computational decontamination tools. Without such precautions, distinguishing true microbial signals from technical noise remains challenging, and interpretation of low-biomass microbiota in CCA should therefore be made cautiously.

**Figure 2:**
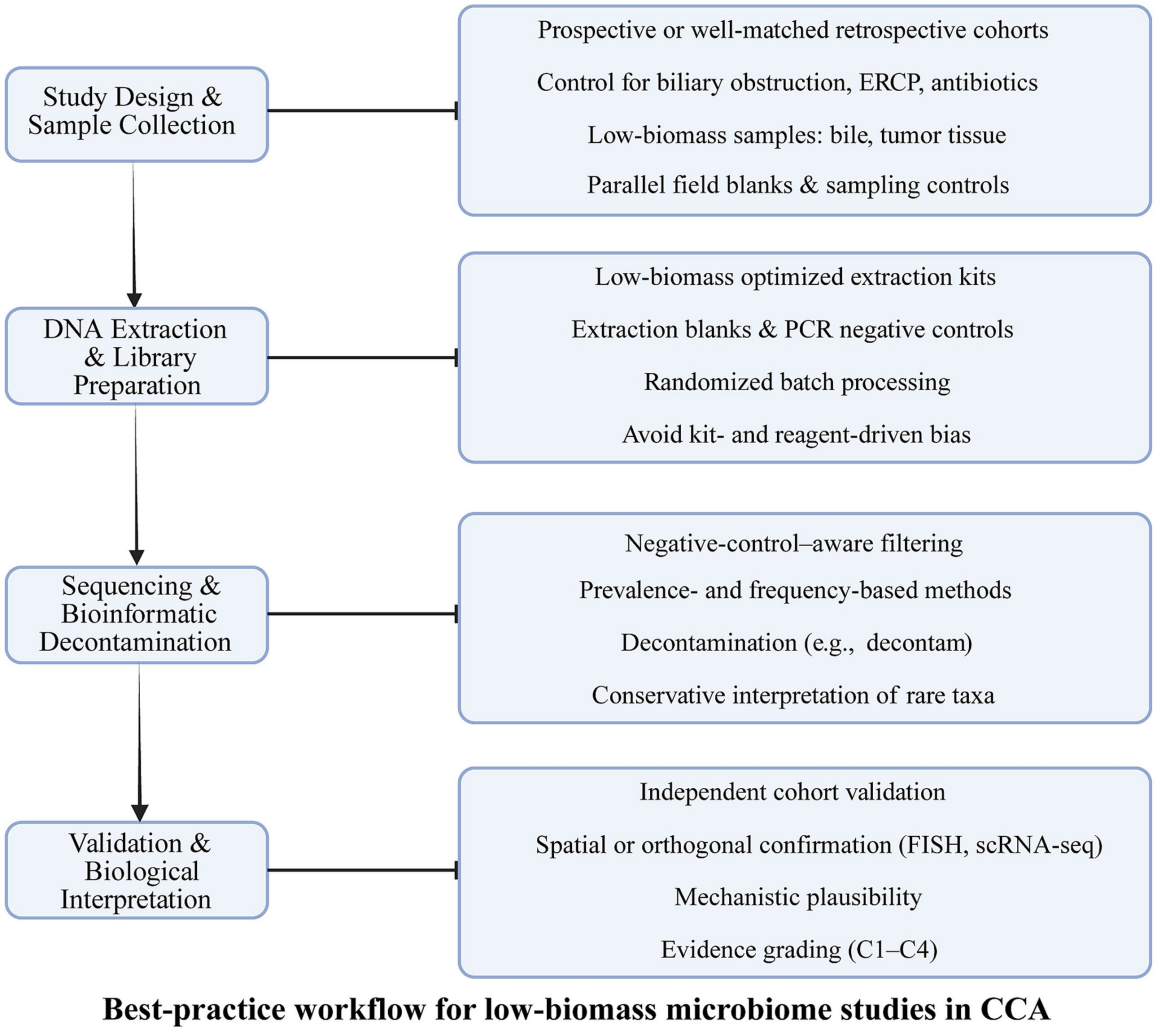
Best-practice workflow for low-biomass microbiome profiling in CCA. A schematic overview of recommended contamination-aware practices for microbiome studies involving low-biomass samples such as bile and tumor tissue in cholangiocarcinoma. The workflow highlights critical steps across study design and sample collection, DNA extraction and library preparation, sequencing and bioinformatic decontamination, and downstream validation and interpretation. Emphasis is placed on the use of parallel negative controls, batch-aware processing, statistical identification of contaminant taxa, and orthogonal validation strategies to improve robustness and biological interpretability of microbiome signals in CCA.

To address the intrinsic challenges of low-biomass microbiome profiling in bile and tumor tissues, we additionally introduce a simplified best-practice workflow (Figure 2). This workflow summarizes key contamination-aware steps spanning study design, sample collection, DNA extraction, sequencing, bioinformatic decontamination, and validation. Emphasis is placed on the use of parallel negative controls, batch-aware processing, statistical contaminant identification, and orthogonal validation strategies, in line with current recommendations for low-biomass microbiome studies.

## 4 Microbial dysbiosis in CCA: a subtype-specific view

Microbiome findings in CCA must be interpreted within the context of its highly heterogeneous pathological background. The anatomical location of CCA and its etiological background (parasite-associated vs. non-parasite-associated) determine its unique microecological characteristics. To more rigorously assess the quality and interpretability of these studies, this review employs a tailored Evidence Grading Framework (Table 2). This framework, which draws on widely accepted standards in the microbiome field for distinguishing correlation from causation and integrates modern epidemiologic approaches such as Mendelian randomization, is essential for evaluating causal strength (Wade and Hall, 2019; Metwaly et al., 2025). The goal is to provide readers with a systematic tool to gage the robustness of available evidence and to prevent purely associative signals (C1 level) from being weighted equivalently to results supported by mechanistic or causal inference (C3 or C4 levels). Given the low-biomass nature of bile and intratumoral samples, most available studies currently fall into C1 level observational evidence, which we explicitly denote to avoid overinterpretation. Accordingly, this chapter will systematically review microbial dysbiosis across different CCA subtypes and ecological niches, integrating this framework to clearly distinguish association from causality evidence.

**Table 2:** Evidence grading framework for CCA microbiome research: distinguishing correlation from causality.

Evidence level (level)	Definition	Typical research methodology	Conclusive evidence
C4 (Mechanistic Validation)	Validates the causal role of a specific microbe through functional experiments.	Germ-Free animal models, Fecal Microbiota Transplantation (FMT), organoid co-culture.	Causality, the strongest evidence.
C3 (Causal Inference)	Uses non-experimental methods to infer the causal relationship between exposure and outcome.	Mendelian Randomization (MR), longitudinal cohort studies.	Causal Association.
C2 (Intervention Evidence)	Assesses the impact of microbial intervention on patient clinical or molecular indicators.	Probiotic/ clinical trials	Clinical Efficacy, though mechanism is often unclear.
C1 (Observational Association)	Reports changes in specific microbial components or metabolites in the disease state.	16S rRNA sequencing, Shotgun Metagenomics, Metabolomics Case–Control studies.	Correlation, the most common level.
C0 (Speculative)	Speculation based on other cancer types or indirect biological evidence.	Analogical studies of mycobiome/virome.	Hypothesis Generation.

### 4.1 Gut microbiome: causal evidence, correlation, and subtype-specific signatures

The gut microbiome indirectly influences biliary health through the enterohepatic circulation and microbial metabolites. Its dysbiosis not only compromises the mucosal barrier and immune homeostasis but also acts as a potential driver of CCA initiation and progression (Ke et al., 2025). This section is organized by the strength of evidence and CCA subtype to provide a systematic and critical evaluation.

#### 4.1.1 High-level evidence: causal inference and mechanistic validation (C3 and C4 level evidence)

A few studies have adopted higher-order methodologies to overcome the limitations of correlation, providing the strongest support for a causal link between the gut flora and CCA.

##### 4.1.1.1 Level C4: mechanistic functional validation

The highest level of evidence comes from animal models. In a bile duct ligation (BDL) model for PSC, researchers used 16S rRNA sequencing to identify an increase in *Clostridiales* and a decrease in *Lactobacillales* following BDL (Fickert et al., 2014; Zhang et al., 2021). Crucially, functional validation revealed that gut dysbiosis compromised the intestinal barrier, leading to the translocation of microbial metabolites like LPS to the liver via the portal vein. LPS subsequently activates the TLR4-CXCL1-CXCR2 axis on hepatocytes, promoting the accumulation of polymorphonuclear myeloid-derived suppressor cells (PMN-MDSCs) in the liver, which drives tumor growth. Antibiotic treatment (Neomycin) effectively suppressed this mechanism, strongly supporting the causal role of the gut microbiome in CCA-like lesions (Zhang et al., 2021). Another pivotal study focusing on ICCA pathogenesis established a detailed causal mechanism: patients exhibited altered gut microbiota and elevated plasma glutamine. Through *in vivo* antibiotic intervention and metabolite supplementation experiments, the researchers confirmed that the gut microbiota alters glutamine metabolism. Mechanistically, glutamine promoted tumor growth by inhibiting ferroptosis via the ALK5/NOX1 axis, a conclusion powerfully demonstrated by the in vivo reversal of ALK5-induced tumor repression upon glutamine addition (Zhang et al., 2024).

##### 4.1.1.2 Level C3: Mendelian randomization causal inference

MR analysis utilizes host genetic variants as instrumental variables to minimize confounding factors, allowing for the inference of a causal effect of gut microbial traits on CCA risk.

An MR analysis focusing on iCCA showed that *Porphyromonadaceae* and *Bacteroidetes* had a protective causal association with iCCA risk, suggesting that the depletion of these taxa may promote iCCA development (Ma et al., 2023). Another MR study further suggested that the gut microbial community may promote or inhibit iCCA progression by regulating host signaling pathways such as AMPK–mTOR and ErbB–EGFR (Chen et al., 2023).

#### 4.1.2 Observational association: subtype-specific dysbiosis (C1 level evidence)

The majority of gut microbiome studies are categorized as C1 level (Observational Association). While these studies cannot establish causality, they are instrumental in providing critical clues for subtype-specific biomarkers. These C1 findings suggest that iCCA and eCCA exhibit distinct microbial shifts that are likely driven by differential anatomical and metabolic pressures. For instance, iCCA is frequently associated with an enrichment of taxa like *Lactobacillus* and *Alloscardovia*, which is correlated with altered conjugated bile acid profiles, indicating a specific bile acid metabolism disorder in the gut-liver axis (Jia et al., 2020). Furthermore, iCCA patients often show elevated levels of pro-inflammatory taxa, such as *Escherichia coli-Shigella*, alongside increases in plasma glutamine, suggesting a potential microbial role in amino acid dysregulation that may inhibit tumor ferroptosis (Zhang et al., 2024). In contrast, eCCA (or BTC) samples frequently report an enrichment of common enteric pathogens like *Klebsiella* and *Gammaproteobacteria,* which is hypothesized to reflect impaired intestinal barrier function and chronic inflammatory states (Ito et al., 2022; Ye et al., 2025). Additionally, in the context of parasite-associated CCA, *Opisthorchis viverrini* infection induces a distinct gastrointestinal dysbiosis, characterized by an increase in *Lachnospiraceae*, *Ruminococcaceae*, and *Lactobacillaceae* and a reduction in *Porphyromonadaceae* and *Erysipelotrichaceae* families (Plieskatt et al., 2013). These subtype-specific C1 observations provide essential molecular signatures that require further high-level causal validation.

The gut microbiome is the most extensively studied ecosystem, yielding widespread Level C1 (Observational Association) evidence for dysbiosis in CCA. Studies consistently report a reduction in overall alpha-diversity and a shared pattern of compositional shifts. This includes the widespread enrichment of pro-inflammatory and barrier-compromising taxa within the Proteobacteria phylum, notably *Klebsiella* and *Escherichia-Shigella*, along with *Enterococcus* (Zhang et al., 2023; Ye et al., 2025). Conversely, beneficial commensals, such as *Bifidobacterium* and *Faecalibacterium*, are significantly depleted (Ye et al., 2025). Notably, the microbial profile of the iCCA subtype shows overlap with cirrhosis and HCC, suggesting that the mechanism in the intrahepatic disease may be heavily dominated by microbial translocation along the gut-liver axis (Jia et al., 2020; Zhang et al., 2021; Table 3).

**Table 3:** Gut microbiome dysbiosis in CCA subtypes and etiologies.

CCA subtype	Significantly altered microbial features	Key metabolite/mechanistic association	Sample grouping	Overall quality assessment	Evidence level	Author year
iCCA	*Lactobacillus*↑, *Alloscardovia*↑	Positively correlated with the plasma/fecal ratio of conjugated bile acids (e.g., GUDCA, TUDCA), suggesting bile acid metabolism disorder	ICC 28, HCC 28, LC 16, HC 12	Exploratory, requires external validation	C1	Jia et al. (2020)
CCA (Associated with PSC/Colitis)	*Lactobacillales*↓ *Actinobacteria*↓ *Clostridiales*↑	Gut barrier dysfunction due to dysbiosis enables LPS translocation, which activates the hepatocyte TLR4/CXCL1 axis to recruit PMN-MDSC, thereby fostering an immunosuppressive environment that promotes tumor growth.	BDL 5, CTR 5* (mice)	Mechanistically validated in animal models; causality supported	C4	Zhang et al. (2021)
iCCA	*Escherichia coli-Shigella* ↑, *Subdoligranulum* ↑	Elevated plasma Glutamine levels. Suggests microbe-mediated amino acid dysregulation, possibly inhibiting Ferroptosis via the ALK5/NOX1 axis	iCCA 26, HC 20	Mechanistically validated in animal models; causality supported	C4	Zhang et al. (2024)
eCCA	*Klebsiella* ↑, *Alistipes* ↑, *Prevotella*↑, *Bifidobacterium*↓	These changes reflect ascending biliary infection or the effect of bile stasis	eCCA 53, HC 21	Exploratory, requires external validation	C1	Ye et al. (2025)
BTC	*Gammaproteobacteria* (mainly Enterobacteriaceae) ↑, *Clostridia* ↓	Reflects impaired intestinal barrier function and enrichment of pro-inflammatory flora	30 BTC, 11 BBD, 10 HC	Exploratory, requires external validation	C1	Ito et al. (2022)
OVa-CCA	*Lachnospiraceae*↑*, Ruminococcaceae*↑, *Lactobacillaceae*↑, *Porphyromonadaceae*↓*Erysipelotrichaceae*↓, *Eubacteriaceae*↓	The microbial and archaeal perturbations co-localized with the flukes may potentiate the distinctive inflammatory response in chronic Opisthorchiasis.	CCA 4, CTR 4* hamsters	Exploratory, requires external validation	C1	Plieskatt et al. (2013)

Studies with sample size <10 are marked with * to indicate exploratory findings with limited statistical power.

### 4.2 Bile microbiome dysbiosis: subtype-specific signatures and critical challenges

The biliary tract (bile and bile duct tissue) is the primary site where microbes directly act on epithelial cells. Emerging evidence challenges the traditional notion of sterile bile, but research in this ecological niche faces inherent challenges related to low biomass, sample contamination, and invasive sampling. Collectively, current studies of the bile microbiome in CCA predominantly provide Level C1 observational evidence, indicating compositional alterations rather than established causal mechanisms.

#### 4.2.1 Subtype-specific signatures of biliary microbiome dysbiosis

Multiple studies using 16S rRNA sequencing have characterized biliary microbiome alterations in CCA. Overall, CCA bile exhibits reduced *Firmicutes* and increased *Bacteroidetes*, with consistent enrichment of genera such as *Enterococcus, Streptococcus*, and *Klebsiella* (Saab et al., 2021). A small 16S rDNA study of bile from BTC patients (*n* = 4) versus benign controls (*n* = 3) showed a clear enrichment of *Enterobacteriaceae* in BTC, indicating a shift toward gut-derived, pro-inflammatory taxa (Ito et al., 2022). Subtype specific analyses have revealed additional differences. In dCCA, Chen et al. reported increased abundances of several rare phyla (including *Gemmatimonadetes* and *Planctomycetes*) and enrichment of *Staphylococcus* and Corynebacterium (Chen et al., 2019). Li et al. (2022) further demonstrated that biliary microbiome profiles differ between pCCA and dCCA, identifying distinct LEfSe-derived biomarkers for each subtype. In PSC-associated and hilar CCA, bile samples show increased species richness and expansion of *Fusobacteria*, suggesting inflammation-associated ecological shifts (Miyabe et al., 2022). Alterations in bile metabolites, such as elevated isoleucine, may further interact with dysbiosis to promote tumorigenesis (Lee et al., 2024). The patterns of biliary dysbiosis exhibit strong subtype specificity. Tumors in the lower biliary tract, such as dCCA/pCCA, primarily reflect ascending colonization of enteric origin (enriched with *Enterobacteriaceae*, *Streptococcus*, etc.). PSC-associated hilar CCA, conversely, may focus more on inflammation-driven dysbiosis (enriched with *Fusobacterium*).

#### 4.2.2 Associative evidence for biliary metabolites

Microbiome-metabolomics studies reveal altered metabolites in CCA bile, providing clues for future C4 mechanistic research. Amino Acid Dysregulation: Studies found significantly elevated Leucine levels in non-CCA cases. Further *in vitro* experiments indicated that Isoleucine exhibits anti-tumor activity. This suggests that biliary microbiome dysbiosis may impact CCA cell proliferation by altering branched-chain amino acid metabolism (Lee et al., 2024; Table 4).

**Table 4:** Bile microbiome dysbiosis in CCA subtypes and etiologies.

CCA subtype	Key findings (vs. control group)	Subtype-specific flora (biomarkers)	Sample grouping	Overall quality assessment	Evidence level	Author year
dCCA	Significant enrichment of rare phyla like *Gemmatimonadetes* and *Nitrospirae.*	*Staphylococcus*↑, *Okibacterium*↑, *Corynebacterium*↑	dCCA 8, CH 60	Exploratory, low-biomass, requires validation	C1	Chen et al. (2019)
pCCA	Exhibits a unique microbial signature compared to gallstone controls.	Top 3 Biomarkers: *Pseudomonas*↑, *Sphingomonas*↑, *Halomonas*↑	pCCA 14, dCCA 9, PC 8, CH 22	Exploratory, low-biomass, requires validation	C1	Li et al. (2022)
dCCA	Significant differences in microbial composition compared to gallstone controls.	Top 3 Biomarkers: *Streptococcus*↑, *Prevotella*↑, *Halomonas*↑	pCCA 14, dCCA 9, PC 8, CH 22	Exploratory, low-biomass, requires validation	C1	Li et al. (2022)
Mainly Hilar CCA (PSC-Associated)	Increased species richness, positively correlated with PSC disease duration.	*Fusobacterium* ↑, highly associated with inflammation-driven progression from PSC to CCA.	PSC 32, CCA (wPSC) 23, CCA (woPSC) 26, CH 17	Exploratory, low-biomass, requires validation	C1	Miyabe et al. (2022)
CCA (Overall)	Enrichment of enteric genera like *Enterococcus* and *Klebsiella* is common in bile	*Enterococcus*↑, *Streptococcus*↑, *Bacteroides*↑, *Klebsiella*↑	CCA 28, CH 47	Exploratory, low-biomass, requires validation	C1	Saab et al. (2021)
CCA (Overall)	Isoleucine In vitro suppressed CCA cell proliferation	*Escherichia coli*↑ Isoleucine↓	CCA 11, Non-CCA 13	Exploratory, low-biomass, requires validation	C3	Lee et al. (2024)
BTC	Indicating a shift toward gut-derived, pro-inflammatory taxa	*Enterobacteriaceae*↑	BTC 4, BBD 3 *	Very small exploratory cohort; insufficient statistical power	C1	Ito et al. (2022)

Studies with sample size <10 are marked with * to indicate exploratory findings with limited statistical power.

#### 4.2.3 Critical assessment of biliary microbiome studies

The clinical value of biliary microbiome studies is limited by their inherent technical and ethical challenges. Insufficient Sample Size and Low Statistical Power: Bile samples are typically obtained via invasive procedures (e.g., ERCP or surgery), resulting in extremely small sample sizes for the partial studies. To avoid overinterpretation, studies with extremely small sample sizes (e.g., bile BTC *n* = 4, BBD *n* = 3) were explicitly labeled as exploratory and assigned lower evidence weight in this review. The reliability and generalizability of the C1 associative results reported on these statistically underpowered datasets must be strictly questioned (Fierer et al., 2025).

High Risk of Contamination: The low biomass of the biliary microbial community makes it highly susceptible to contamination during sampling. Whether collected retrogradely via ERCP through the duodenum or intraoperatively, there is a risk of introducing oral or enteric flora (Al-Kabban et al., 2025). This confounding severely limits our ability to distinguish true colonizers from environmental contaminants. Lack of Healthy Controls: Due to the inherent risks of invasive procedures like ERCP (infection, bleeding, pancreatitis), it is ethically and safety-wise near impossible to recruit healthy individuals as controls for a normal biliary microbiome. This makes it difficult for studies to definitively establish whether changes in the disease state are true “dysbiosis” or merely individual variation or sampling bias. Future research must overcome these challenges by employing stringent negative controls, large-scale multi-center sampling, and high-resolution metagenomics to advance biliary microbial biomarkers toward higher Level validation.

### 4.3 Intratumoral microbiome: etiological and prognostic signatures

The microbial community within the tumor tissue and TME has been shown to be a critical component in CCA pathogenesis, progression, and therapeutic response. Compared to the bile and gut flora, the intratumoral microbiome more directly reflects local microecological changes, though its analysis also faces challenges of extremely low biomass and high spatial heterogeneity (Selway et al., 2020). Evidence from intratumoral microbiome studies in CCA remains largely Level C1, reflecting association-based findings without functional validation.

#### 4.3.1 Subtype specificity: differentiation by etiology and anatomical location

The composition of the tumor tissue microbiome strictly depends on the CCA’s etiology and anatomical location, rather than a generic CCA signature. Clear subtype-specific microbial differences are evident across both etiological and anatomical categories of CCA. In *Opisthorchis viverrine*-associated CCA (OVa-CCA), tumor tissues show significant enrichment of *Bifidobacteriaceae* and *Enterobacteriaceae*, whereas non-parasitic CCA (nOVa-CCA) is characterized by increased *Stenotrophomonas*, consistent with its known association with biliary infection (Pérez et al., 2014; Chng et al., 2016). These findings suggest that parasite-driven and non–parasite-driven CCA likely arise through distinct microbiome-mediated mechanisms, further supported by functional predictions indicating greater bile acid and ammonia production potential in OVa-CCA (Chng et al., 2016). Anatomical subtype analyses demonstrate additional divergence: in large iCCA cohorts (*n* = 121), *Firmicutes, Actinobacteria, and Bacteroidetes* are enriched within tumor tissue, while adjacent normal tissue is dominated by *Proteobacteria* (Xin et al., 2024). In contrast, eCCA specimens show enrichment of *Helicobacter pylori* and its virulence genes (cagA, vacA), alongside elevated *Fusobacterium* and *Prevotella* (Boonyanugomol et al., 2012; Avilés-Jiménez et al., 2016); however, given that eCCA samples are typically obtained via ERCP, potential contamination from oral or gastric microbiota must be carefully considered.

#### 4.3.2 Evidence grading: from association to mechanism and prognosis

**Figure 3:**
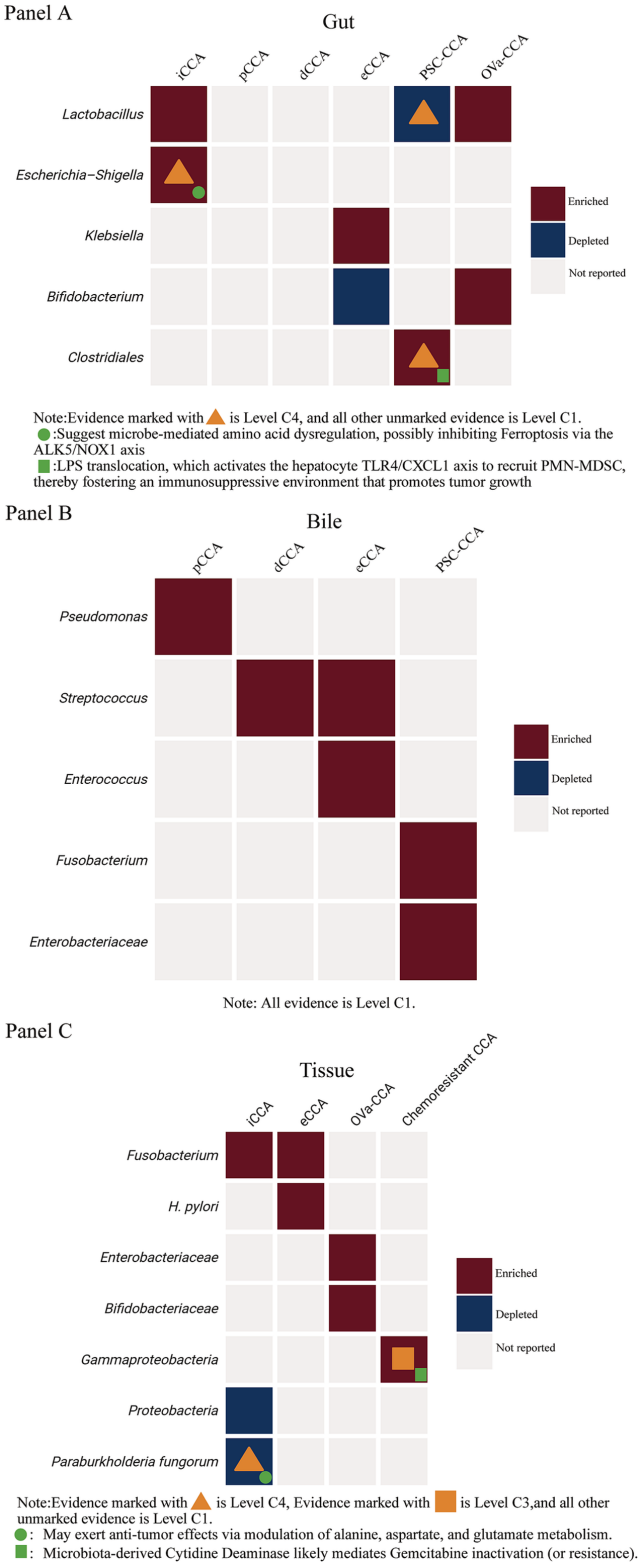
Integrated overview of reported microbiome alterations across CCA subtypes and anatomical niches. Panel A (Gut). Heatmap illustrating gut microbiota alterations across CCA subtypes, including iCCA, pCCA, dCCA, eCCA, PSC-associated CCA, and OVa-CCA. Taxa are shown as enriched (red), depleted (blue), or not reported (light gray). Evidence strength is indicated by symbols (▲, C4; unmarked, C1). Mechanistic annotations denote inferred microbiota-driven amino acid dysregulation affecting ferroptosis (●) or LPS-mediated immune modulation (■). Panel B (Bile). Heatmap showing bacterial taxa reported as enriched (red), depleted (blue), or not reported (light gray) in bile samples from pCCA, dCCA, eCCA, and PSC-associated CCA. Unless otherwise indicated, evidence is derived from cross-sectional human studies (C1). Panel C (Tissue). Heatmap summarizing tumor tissue–associated microbiome alterations across iCCA, eCCA, OVa-CCA), and chemoresistant CCA. Evidence strength is indicated by symbols: ▲ C4 (mechanistic validation), ■ C3 (functional human studies), unmarked cells C1. Mechanistic annotations denote anti-tumor metabolic effects (●) or microbiota-mediated gemcitabine inactivation (■).

Recent intratumoral microbiome studies extend beyond simple associative findings (Level C1) and now provide emerging mechanistic insights (Level C4) as well as prognostic relevance. Single-cell transcriptomics and FISH have confirmed the presence of bona fide bacteria within CCA tumors, enabling functional interrogation of specific taxa. For example, *Paraburkholderia fungorum* was found to be enriched in peritumoral regions and inversely correlated with CA19-9 levels, and *in vitro* experiments demonstrated that it may exert anti-tumor effects by modulating alanine, aspartate, and glutamate metabolism, representing Level C4 mechanistic evidence (Chai et al., 2023). Similarly, *gamma-Proteobacteria* have been identified as markedly enriched in gemcitabine and cisplatin resistant CCA tissues, accompanied by elevated intratumoral metabolites such as acetylcholine and adenine. This pattern mirrors the mechanism described in pancreatic cancer, in which *gamma-Proteobacteria* express cytidine deaminase to inactivate gemcitabine, suggesting a microbe-driven pathway of chemotherapy resistance in CCA as well (Choy et al., 2018; Sitthirak et al., 2022). Beyond mechanistic insights, intratumoral diversity also carries prognostic value: in iCCA, higher bacterial alpha-diversity within tumor tissue has been associated with shorter overall and recurrence-free survival, providing Level C1 evidence that the intratumoral microbiome may serve as a prognostic biomarker (Xin et al., 2024; Table 5). To provide an integrated overview, we constructed a multi-panel comparative heatmap (Figure 3) summarizing subtype-specific microbiome enrichment or depletion across gut, bile, and tumor tissue, together with the corresponding levels of evidence and reported functional or metabolic features.

**Table 5:** Associations of tissue microbiome dysbiosis in CCA: prognosis and mechanistic clues.

CCA subtype	Significantly altered microbial features	Key metabolite/mechanistic association	Sample grouping	Overall quality assessment	Evidence level	Author year
eCCA vs. Benign Biliary Diseases	*Nesterenkonia*↓, *Methylophilaceae*↑, *Fusobacterium*↑, *Prevotella*↑, *Actinomyces*↑, *Novosphingobium*↑, *H. pylori*↑	Confirmed presence of *H. pylori* virulence genes (*cagA*, *vacA*).	eCCA 10, BBP 10	Exploratory, low-biomass, requires validation	C1	Avilés-Jiménez et al. (2016)
OVa-CCA vs. nOVa-CCA (Etiological)	*OVa: Bifidobacteriaceae↑, Enterobacteriaceae↑. nOVa: Stenotrophomonas↑.*	OVa-microbiome suggested to have greater potential for bile acid and ammonia production, implicating these in carcinogenesis.	CCA (OVa) 28, CCA (nOVa) 32, N (OVa) 28, N (nOVa) 32	Exploratory, low-biomass, requires validation	C1	Chng et al. (2016)
ICCA Tissue (Spatial/Functional)	*Proteobacteria*↓, *Armatimonadetes*↑, *Verrucomicrobia*↑, *Fusobacterium*↑	*Paraburkholderia fungorum* (paracancerous ↑) exerts anti-tumor effects via modulation of alanine, aspartate, and glutamate metabolism. (Spatial context confirmed by FISH/scRNA-seq).	ICCA 45, N 49	High-quality mechanistic study with spatial validation	C4	Chai et al. (2023)
CCA (Chemoresistance)	*Gammaproteobacteria* ↑, acetylcholine↑, adenine↑, carnitine↑, inosine↑	*gamma-Proteobacteria* expression of Cytidine Deaminase (mechanism for Gemcitabine inactivation).	CCA 36	Exploratory, low-biomass, requires validation	C3	Sitthirak et al. (2022)
iCCA (Prognosis)	*Firmicute*↑, *Actinobacteri*↑, *Bacteroidete*↑, *Acidobacteria*↑, *Proteobacteria*↓	Higher intratumoral alpha-diversity associated with shorter OS and RFS	ICCA 121, PT 89	Exploratory, low-biomass, requires validation	C1	Xin et al. (2024)

#### 4.3.3 Assessment of research quality and sample size

The quality of tumor tissue microbiome studies remains heterogeneous, underscoring the need to carefully contextualize research findings. Although a few studies have achieved relatively large sample sizes, most functional and spatial analyses are derived from much smaller cohorts. For these smaller datasets, C1-level associative findings should be interpreted primarily as hypothesis-generating rather than definitive, and any proposed C4-level mechanistic insights will require validation in larger, independent cohorts. Notably, the spatially resolved work by Chai et al. demonstrated that bacteria inhabit distinct tumor cell populations, providing critical spatial context for future mechanistic studies and helping to distinguish true intratumoral colonization from potential contamination (Chai et al., 2023).

### 4.4 Oral and blood microbiome dysbiosis: systemic and non-invasive biomarker potential

The oral and blood microbiomes—representing systemic ecological niches—are emerging areas of investigation in CCA, although current evidence remains limited and uniformly at Level C1 (observational association). Alterations in these niches may mirror systemic inflammation and immune dysregulation, offering potential for non-invasive biomarker development. Oral microbiome perturbations may influence CCA through microbial translocation, either via ingestion and subsequent gut colonization or through hematogenous spread to the liver. Enrichment of pro-inflammatory taxa such as *Fusobacteriota*, well known for their carcinogenic roles in gastrointestinal tumors, supports the speculative notion (Level C0) that oral dysbiosis may indirectly shape the inflammatory microenvironment of CCA (Rao et al., 2022). In parallel, the blood microbiome—particularly microbial DNA encapsulated within extracellular vesicles (EVs)—has gained interest as a stable circulating biomarker, as EVs protect microbial nucleic acids from rapid host clearance and may thus provide a promising avenue for non-invasive CCA detection (Lee et al., 2020; Table 6).

**Table 6:** C1 level observational associations of microbiome dysbiosis in non-invasive samples (Saliva and plasma extracellular vesicles).

Sample and data source	Sample grouping	Sequencing methods	Dysbiosis in the microbiome and metabolites	Overall quality assessment	Evidence level	Author year
Saliva samples	CCA 74, HCC 35, HC 150	16S rDNA sequencing	*Cyanobacteria*↑, *Spirochaetota*↑, *Campilobacteria*↑, *Fusobacteriota*↑, *Firmicutes*↑, *Synergistota*↑, *Desulfobacterota*↑, *Chloroflexi*↑, *Actinobacteria*↓, *Bacteroidota*↓,	Exploratory, low-biomass, requires validation	C1	Rao et al. (2022)
Extracellular vesicles in plasma	BTC 24, BBP 43, HC 88	16S rDNA sequencing	*Bifidobacteriaceae* family↑, *Oxalobacteraceae Ralstonia* ↑, *Pseudomonaceae* family↓, *Corynebacteriaceae Corynebacterium*↓, *Comamonadaceae Comamonas* species↓	Exploratory, low-biomass, requires validation	C1	Lee et al. (2020)

A recent study reported a blood microbiome–based diagnostic model for CCA with an “AUC of 1.0” (Lee et al., 2020), but such findings warrant careful scrutiny. The study’s small sample size makes the perfect performance highly susceptible to overfitting, suggesting that the model may reflect memorization of sample-specific signals or batch effects rather than true biological discrimination. Moreover, given the extremely low biomass of circulating microbial DNA and the high risk of environmental contamination, these C1-level observations require validation through larger, multi-center cohorts with independent external testing before any clinical translation can be considered (Wade and Hall, 2019).

### 4.5 Non-bacterial microbiome: fungi, viruses, and archaea

The human microbiome is not limited to bacteria; fungi, viruses (including bacteriophages), and archaea collectively constitute a complex, multi-kingdom microecological system that may influence carcinogenesis through immune, metabolic, and ecological interaction (Lange and Ramirez, 2021). While research on the tumor mycobiome and virome has expanded rapidly in several gastrointestinal malignancies, investigations into non-bacterial microbial components in CCA remain scarce. Nevertheless, accumulating indirect evidence suggests that non-bacterial dysbiosis represents a biologically plausible and clinically relevant, yet underexplored, dimension of CCA pathogenesis.

To provide a structured overview, available evidence for fungal, viral, and archaeal involvement in hepatobiliary and related gastrointestinal cancers is summarized in Table 7, including extrapolated findings from biologically analogous malignancies.

**Table 7:** Summary of non-bacterial microbiome components studies relevant to hepatobiliary cancers and related malignancies.

Kingdom	Cancer type	Sample	Sample grouping	Key findings	Overall quality assessment	Evidence level	Author year
Fungi	iCCA	Feces	ICCA 23, HC 17	*Candida albicans*↑, *Saccharomyces*↓	Exploratory, requires external validation	C1	Zhang et al. (2022)
Fungi	PDAC	Tissue	Not reported	Malassezia spp.↑, C3 activation	Mechanistically validated in animal models; causality supported	C4	Aykut et al. (2019)
Virus	CCA	Liver	CCA 7113, CON 24763	HBV/HCV oncogenesis	Meta-analysis And Strong epidemiological evidence	C3	Tan et al. (2020)
Phage	CRC	Feces	Carcinomas 30, Adenomas 30, Healthy 30	Phage-driven dysbiosis	Exploratory, requires external validation	C1	Hannigan et al. (2018)
Archaea	CRC	Feces	CRC 748, Adenoma 471, HC 882 (10 cohorts, 7 countries)	*Methanobacteriota*↑, *Methanomassiliicoccales*↓	Large multicohort, lacks causal or functional validation	C2	Li et al. (2025)

#### 4.5.1 Fungal community (mycobiome)

Direct characterization of the CCA-associated mycobiome is limited; however, emerging data suggest that fungal dysbiosis may contribute to tumor-associated inflammation and immune modulation (Wang et al., 2024; Camille et al., 2026). Zhang et al. reported intestinal fungal alterations in patients with intrahepatic CCA, characterized by enrichment of opportunistic fungi such as *Candida albicans* and depletion of potentially beneficial taxa such as *Saccharomyces cerevisiae* compared with healthy controls, indicating a disease-associated mycobiome imbalance (Evidence level C1) (Zhang et al., 2022).

Mechanistic insights can be cautiously extrapolated from other gastrointestinal cancers. In pancreatic ductal adenocarcinoma, *Malassezia* species translocate to tumor tissue and promote tumor progression through mannose-binding lectin–complement activation, providing strong causal evidence for fungal involvement in tumor biology (Evidence level C3–C4) (Aykut et al., 2019). By analogy, biliary or intestinal fungal dysbiosis—particularly *Candida* overgrowth—may influence CCA development through immune suppression, chronic inflammation, or fungal metabolite–mediated modulation of bile acid signaling (C0–C1 speculation).

Despite this potential relevance, systematic bile- or tissue-based mycobiome studies in CCA are lacking. Fungal profiling is technically challenging due to low fungal biomass, high susceptibility to environmental contamination, and biases associated with internal transcribed spacer (ITS) sequencing and incomplete reference database (Fletcher et al., 2023).

#### 4.5.2 Virome and bacteriophages

The virome represents another understudied component of the CCA microenvironment. Among viruses, HBV and HCV have well-established causal roles in intrahepatic CCA through chronic inflammation, genomic integration, and oncogenic signaling, constituting Level C3 evidence (Tan et al., 2020; An et al., 2022). In contrast, the roles of other oncogenic or latent viruses, such as human papillomavirus (HPV) or cytomegalovirus (CMV), remain speculative and poorly defined in CCA.

Beyond eukaryotic viruses, bacteriophages are increasingly recognized as key regulators of microbial ecology by shaping bacterial composition, virulence, and antibiotic resistance. Phage-mediated modulation of gut microbiota has been implicated in colorectal cancer and inflammatory bowel disease; however, phage communities in bile or tumor tissue have not yet been systematically characterized in CCA. Given the central role of bacteria in biliary inflammation, phage-driven restructuring of microbial networks represents a conceptually promising but unexplored mechanism in CCA pathogenesis (C0) (Petrov et al., 2022).

#### 4.5.3 Archaea

Archaea constitute a minor yet metabolically influential fraction of the gut microbiota, predominantly represented by methanogens such as *Methanobrevibacter*. By consuming hydrogen, archaea regulate bacterial fermentation efficiency and indirectly influence short-chain fatty acid production and intestinal redox balance (Chaudhary et al., 2018).

Although archaeal dysbiosis has not been directly studied in CCA, indirect evidence from gastrointestinal diseases suggests that altered archaeal abundance may modulate bile acid metabolism and immune homeostasis. In the context of CCA, archaeal expansion or depletion could reshape the metabolic microenvironment of the biliary tract by influencing bile acid composition or inflammatory signaling (C0 speculation). The lack of archaeal data in CCA largely reflects technical limitations rather than biological irrelevance.

#### 4.5.4 Kingdom-specific technical and methodological barriers

Characterizing non-bacterial microbiome components poses substantial technical challenges, particularly in low-biomass environments such as bile and tumor tissue (Salter et al., 2014).

##### 4.5.4.1 Fungal profiling challenges

Mycobiome analysis relies primarily on ITS sequencing, which is highly sensitive to contamination and primer bias. Low fungal biomass and incomplete fungal reference databases further limit taxonomic resolution and reproducibility (Fletcher et al., 2023; Avershina et al., 2025).

##### 4.5.4.2 Virome and phage-specific challenges

Virome analysis is hindered by the overwhelming background of host DNA, limited viral reference genomes, and difficulties in distinguishing active infection from latent or passenger viral DNA. Phage annotation and functional inference remain particularly challenging due to rapid viral evolution and sparse databases (Khan Mirzaei et al., 2021; Wu and Peng, 2024). In addition, phage–bacteria interactions require dedicated analytical frameworks, and batch effects can easily generate false-positive fungal or viral signals (Auslander et al., 2020; Tan et al., 2023).

##### 4.5.4.3 Archaeal detection challenges

Standard 16S rRNA primers are biased toward bacteria and frequently fail to capture archaeal sequences. Extremely low archaeal abundance necessitates targeted primers or deep shotgun sequencing, which are rarely applied in CCA studies (Adam et al., 2022).

To overcome these barriers, future studies should adopt multi-kingdom shotgun metagenomics, rigorous negative controls, and analytical pipelines specifically optimized for low-biomass microbial ecosystems (Selway et al., 2020).

#### 4.5.5 Hypothesis-driven links between non-bacterial dysbiosis and CCA pathogenesis

Based on current evidence, we propose several testable hypotheses linking non-bacterial microbiome alterations to CCA biology.

##### 4.5.5.1 Fungal dysbiosis hypothesis

Intestinal or biliary fungal overgrowth, particularly Candida species, promotes CCA progression by inducing immunosuppressive myeloid cell populations and altering bile acid signaling through fungal metabolites.

##### 4.5.5.2 Virome and phage hypothesis

Phage-mediated remodeling of biliary bacterial communities enhances microbial virulence and chronic inflammation, indirectly accelerating biliary epithelial injury and malignant transformation.

##### 4.5.5.3 Archaeal metabolic hypothesis

Altered archaeal abundance reshapes hydrogen flux and bile acid metabolism, creating a metabolic niche that favors immune tolerance and oncogenic signaling in the biliary tract.

These hypotheses provide a conceptual framework for future multi-kingdom microbiome studies and highlight non-bacterial microbes as a promising frontier in CCA research.

## 5 Microbiome-based biomarkers for CCA: models, challenges, and recommendations

### 5.1 Microbiome-based diagnostic models for CCA and their validation status

Emerging evidence indicates that alterations in the gut, bile, and circulation-associated microbiomes are associated with CCA and may provide non-invasive biomarkers for early diagnosis. To date, multiple diagnostic models have been proposed based on fecal microbiota, biliary microbiota, and circulating microbial components; however, these models differ substantially in sample source, feature selection strategies, modeling approaches, and—critically—their degree of validation.

Fecal microbiome–based models represent the most extensively studied approach. In a representative study, Zhang et al. constructed a predictive model based on the relative abundances of *Burkholderia-Caballeronia-Paraburkholderia*, *Faecalibacterium*, and *Ruminococcus_1*, which distinguished CCA patients from healthy controls with a high diagnostic performance (AUC = 0.973), outperforming the conventional biomarker CA19-9 within the same cohort (Zhang et al., 2021). Despite its strong internal performance, this model was derived from a single-center cohort and has not yet undergone independent external validation, limiting its generalizability across populations.

Subsequent studies have explored hybrid models integrating microbial features with established clinical markers. For example, Zhang et al. demonstrated that combining CA19-9 with the *Bifidobacterium*/*Klebsiella* (B/K) ratio yielded an exceptionally high AUC of 0.999 for CCA discrimination (Zhang et al., 2023). While this finding highlights the potential complementary value of microbiome-derived features, the model was similarly developed in a limited cohort without external validation, raising concerns regarding overfitting and population-specific effects.

Beyond fecal samples, alternative biospecimens have also been investigated. Lee et al. profiled microbial DNA within plasma-derived extracellular vesicles and developed a diagnostic model based on *Bifidobacteriaceae*, *Pseudomonadaceae*, and the genera *Corynebacterium*, *Ralstonia*, and *Comamonas*, reporting an AUC of 1.0 (Lee et al., 2020). However, this study relied on a relatively small exploratory cohort and lacked validation in independent populations, underscoring the need for cautious interpretation despite its impressive performance metrics.

Bile microbiome–based models offer a more disease-proximal diagnostic source and have demonstrated robust discriminatory ability between CCA and benign biliary diseases, with reported AUC values reaching up to 0.96 in some studies (Wang et al., 2025). Nevertheless, most bile-based models remain confined to single-center designs and exhibit considerable heterogeneity in sampling procedures, sequencing platforms, and analytical pipelines, with standardized cut-off values and external validation still largely absent.

To enable systematic comparison, we summarize representative microbiome-based diagnostic models for CCA in Table 8, detailing sample type, cohort size, microbial feature level, modeling strategy, diagnostic performance, and validation status. Across studies, a consistent pattern emerges: while many models achieve excellent internal diagnostic accuracy, the vast majority lack multi-center external validation and prospective evaluation, which remain essential prerequisites for clinical translation. Furthermore, differences in cohort composition—including geographic origin, dietary background, and the inclusion of comorbid conditions such as primary sclerosing cholangitis—further complicate cross-study comparability (Kori et al., 2024).

**Table 8:** Reported microbiome-based diagnostic models for CCA.

Author year	Sample type	Cohort size (CCA/control)	Feature level	Model/method	AUC (95% CI)	Validation	Quality rating
Lee et al. (2020)	Extracellular vesicles in plasma	BTC 24, BBP 43, HC 88	*Bifidobacteriaceae*, *Pseudomonadaceae*, *Corynebacterium*, *Ralstonia*, *Comamonas*	Logistic regression prediction model including age/sex	1.000 (0.8518–1.000)	Internal validation (training/ validation split)	Moderate
Zhang et al. (2021)	Gut microbiome	53 CCA / 40 HC	Genus (B-F-R model)	Random Forest	0.973 (0.932–1.00)	Internal (train/test)	Moderate
Zhang et al. (2023)	Gut microbiome	42 CCA / 16 HC	Bifidobacterium / Klebsiella ratio	Ratio index	Not reported	No external validation	Low
Wang et al. (2025)	Bile microbiome	266 total (42 new cohort + others)	Genera signatures (Biletypes)	Random Forest	0.931	External validation	High

Collectively, current microbiome-based diagnostic models for CCA demonstrate promising potential but are best regarded as proof-of-concept frameworks rather than clinically deployable tools. Future efforts must prioritize standardized study designs and independent validation to establish robust, generalizable biomarkers suitable for early detection in diverse patient populations.

### 5.2 Biomarker stability and cross-population heterogeneity

A crucial concern for clinical translation is the stability and generalizability of microbiome-based biomarkers across populations. Microbiome composition is shaped by host factors such as ethnicity, diet, geography, antibiotic exposure, and comorbidities (Yatsunenko et al., 2012; Patangia et al., 2022; Xiao et al., 2024). Consequently, models developed in one cohort may not generalize to other demographic groups without recalibration. For example, gut microbiota signatures derived from CCA patients in East Asian cohorts may differ fundamentally from those in Western cohorts due to distinct baseline microbial community structures (Fu et al., 2025).

Technical factors also contribute to inter-study variability, including differences in DNA extraction kits, choice of 16S rRNA variable regions, sequencing depth, and bioinformatic processing (Fierer et al., 2025). These pre-analytical and analytical variations can lead to batch effects that overshadow true biological signal, reducing biomarker reproducibility.

Disease heterogeneity within CCA itself — including intrahepatic versus extrahepatic subtypes, presence of PSC, and differences in tumor stage — further complicates biomarker stability (Harrison and Visser, 2024; Fu et al., 2025). It is therefore imperative that diagnostic models incorporate robust adjustment for confounding variables and are validated across diverse geographic and clinical contexts before clinical application.

### 5.3 Recommendations for standardizing microbiome-based biomarker discovery pipelines

To address the challenges above and enhance the clinical applicability of microbiome-based biomarkers in CCA, we propose the following standardized pipeline for biomarker discovery and validation:

*Cohort design and metadata harmonization*: Assemble multi-center and multi-ethnic cohorts with balanced representation of CCA subtypes and relevant controls (benign biliary disease, PSC, healthy). Define standardized inclusion/exclusion criteria and collect comprehensive metadata (diet, medication history, comorbidities).
*Sample collection and processing*: Utilize consistent protocols for sample handling (e.g., bile and stool collection), include negative extraction controls, and implement rigorous contamination control measures for low-biomass specimens. Adopt harmonized sequencing strategies (e.g., common 16S rRNA regions or shotgun metagenomics) and document batch information.
*Feature selection and model development*: Apply robust dimensionality reduction and cross-validation strategies to avoid overfitting. Where feasible, integrate multi-omic features (microbiome, metabolome, bile acids) to improve sensitivity and specificity.
*External validation and calibration*: Validate models in independent cohorts distinct from the discovery set. Report calibrated thresholds and performance metrics (AUC, sensitivity, specificity with confidence intervals) stratified by cohort.
*Clinical benchmarking*: Compare microbiome-based models against existing clinical biomarkers (e.g., CA19-9) and imaging findings to define incremental diagnostic value.

By adopting a rigorous and standardized pipeline, the field can move beyond exploratory models toward clinically actionable microbiome-based diagnostic tools capable of improving early detection in high-risk populations (Kim et al., 2017; Bokulich et al., 2020; Kori et al., 2024).

## 6 Mechanistic pathways linking microbiota to cholangiocarcinogenesis

**Figure 4:**
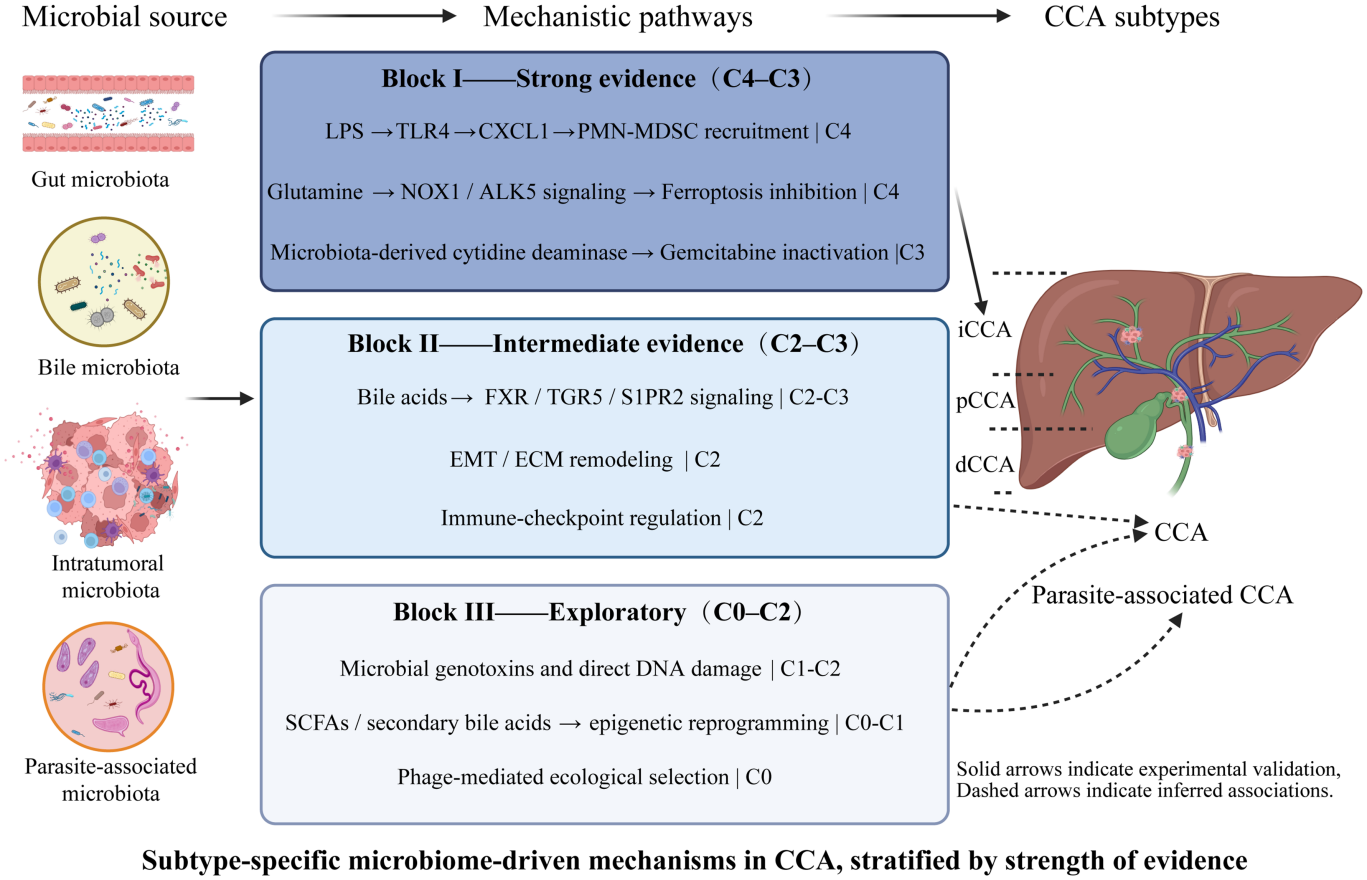
Subtype-specific microbiome-driven mechanisms in cholangiocarcinoma, stratified by strength of evidence. Color intensity indicates strength of evidence (C1–C4). Solid arrows indicate experimental validation. Dashed arrows indicate inferred associations. Mechanisms are annotated with predominant CCA subtype.

Microbial dysbiosis likely promotes cholangiocarcinogenesis through multiple, partially overlapping mechanisms that vary by anatomical and etiologic subtype. To avoid overgeneralization, the following mechanisms are discussed with explicit reference to their predominant subtype relevance (iCCA, eCCA, or parasite-associated CCA) and the highest level of supporting evidence available. For clarity, mechanisms are discussed in an order reflecting both biological continuity and strength of supporting evidence, with higher-confidence, subtype-specific pathways presented first, followed by context-dependent and hypothesis-generating mechanisms (Figure 4).

### 6.1 Block I. High-confidence, subtype-specific mechanisms with experimental validation (C4–C3)

#### 6.1.1 Inflammation-driven immune remodeling (TLR4 → CXCL1 → MDSC)

##### 6.1.1.1 Subtype relevance: predominantly iCCA

###### 6.1.1.1.1 Highest level of evidence: C4 (validated in murine models)

Microbe-derived ligands such as LPS activate pattern recognition receptors (e.g., TLR4) on hepatocytes and cholangiocytes, inducing chemokines (e.g., CXCL1) that recruit immunosuppressive myeloid populations (PMN-MDSCs), thereby creating a tolerogenic niche that favors tumor growth. Experimental work in iCCA models demonstrates microbe-to-hepatocyte signaling that drives MDSC accumulation via TLR4–CXCL1, providing a mechanistic template likely relevant to CCA in contexts of barrier breach or cholangitis (Zhang et al., 2021). This mechanism is supported by C4-level experimental validation in iCCA models.

### 6.2 Ferroptosis regulation and metabolic reprogramming (ALK5/NOX1)

#### 6.2.1 Subtype relevance: iCCA

##### 6.2.1.1 Highest level of evidence: C4 (mechanistic mouse models)

Microbiome-dependent modulation of amino-acid metabolism (notably glutamine) can influence ferroptosis susceptibility through pathways involving ALK5 and NOX1. Recent mechanistic work in intrahepatic CCA links gut microbiota–altered glutamine metabolism to inhibition of ferroptosis via the ALK5/NOX1 axis, implicating microbial-metabolic regulation as a cell-death escape mechanism. This axis provides a direct example of how microbial metabolic effects can alter tumor cell vulnerability (Zhang et al., 2024). Importantly, this pathway has so far been demonstrated specifically in iCCA models, and its relevance to extrahepatic CCA remains unknown.

### 6.3 Chemotherapy resistance via microbial drug metabolism

#### 6.3.1 Subtype relevance: iCCA and eCCA

##### 6.3.1.1 Highest level of evidence: C3 (functional human tumor analyses)

Intratumoral or biliary bacteria can enzymatically inactivate chemotherapeutic agents. A well-documented example from pancreatic cancer shows *Gammaproteobacteria* expressing long-form cytidine deaminase (CDD_L) that degrades gemcitabine, thereby mediating drug resistance; analogous findings of *Gammaproteobacteria* enrichment in chemo-resistant CCA tissues and metabolomic shifts suggested a potentially conserved microbe-driven resistance mechanism that requires direct validation in CCA.(Choy et al., 2018; Sitthirak et al., 2022). Direct functional validation of microbial drug metabolism in CCA remains limited and warrants targeted investigation.

### 6.4 Block II. Context-dependent metabolic and stromal mechanisms (C2–C3)

#### 6.4.1 Bile-acid receptor signaling (FXR, TGR5, S1PR2) — a metabolic–inflammatory axis

##### 6.4.1.1 Subtype relevance: eCCA > iCCA

###### 6.4.1.1.1 Highest level of evidence: C2–C3

Accumulating evidence indicates that microbiome-driven alterations in bile acid composition act as key signaling mediators linking dysbiosis to cholangiocarcinogenesis. In cholestatic conditions frequently observed in CCA, impaired bile flow disrupts enterohepatic circulation, resulting in the accumulation of specific primary and secondary bile acids that activate nuclear and G-protein–coupled receptors, including FXR, TGR5, and S1PR2 (Wang et al., 2025; Wu et al., 2025).

Secondary bile acids such as deoxycholic acid (DCA), generated through bacterial 7α-dehydroxylation, have been shown to promote hepatic inflammation, oxidative DNA damage, fibrosis, and senescence-associated secretory phenotypes in stromal cells, thereby creating a tumor-promoting microenvironment (Ridlon et al., 2013; Yoshimoto et al., 2013). Conversely, primary bile acids can exert anti-tumor immune effects by inducing CXCL16-mediated recruitment of CXCR6^+^ NKT cells; however, this protective axis is suppressed by microbiota-derived secondary bile acids (Ma et al., 2018).

In cholestatic models relevant to CCA, conjugated primary bile acids (e.g., taurocholate) activate S1PR2, a receptor highly expressed in cholangiocytes and CCA cells, driving proliferative and pro-survival signaling through ERK/AKT and Hippo–YAP pathways. Genetic deletion of S1PR2 in bile duct ligation models attenuates cholangiocyte proliferation, inflammation, and fibrosis, supporting a mechanistic link between bile acid signaling and biliary pathology (Nagahashi et al., 2016; Liu et al., 2018).

Importantly, although these pathways establish a biologically coherent microbiome–bile acid–receptor signaling axis integrating metabolic dysregulation, immune modulation, and stromal activation, most supporting evidence derives from cholestatic liver injury models, hepatocellular carcinoma, or observational human studies rather than microbiota-controlled CCA systems. Meanwhile, it is important to note that most supporting evidence establishes causality at the level of bile acid–receptor signaling rather than at the level of microbiota-specific exposure. Direct experimental studies demonstrating that targeted microbial manipulation drives these receptor-mediated effects in CCA models remain limited.

Accordingly, this mechanism is classified as C2–C3 evidence, reflecting strong pathway-level causality supported by experimental models, but incomplete microbiota-specific causal validation.

### 6.5 EMT, stromal remodeling and fibro-inflammatory signaling

#### 6.5.1 Subtype relevance: iCCA predominant

##### 6.5.1.1 Highest level of evidence: C2

Microbial products such as lipopolysaccharide and dysbiosis-associated metabolites can amplify chronic inflammation and activate profibrotic signaling pathways, including TGF-*β*, NF-κB, and Wnt/β-catenin, in both cholangiocytes and stromal cells. These pathways promote epithelial–mesenchymal transition (EMT), cancer-associated fibroblast activation, and extracellular-matrix deposition—hallmarks of the dense, desmoplastic stroma characteristic of many iCCA tumors (Chen et al., 2023; Sun et al., 2024; Klungsaeng et al., 2025).

Recent evidence suggests that excessive bile acids can directly activate GPBAR1 on cancer-associated fibroblasts, inducing CXCL10 expression and fostering EMT, metastatic dissemination, neutrophil recruitment, and immune suppression within the tumor microenvironment (Huang et al., 2025). Analogous pathogen-associated EMT mechanisms have been well documented in other gastrointestinal malignancies (Kubo et al., 2025; Li et al., 2025), further supporting the biological plausibility of microbiota-driven stromal remodeling in CCA.

Nevertheless, most available data are indirect, relying on correlations between dysbiosis, inflammation, fibrosis, and EMT signatures, or extrapolation from related GI cancers. Direct experimental evidence demonstrating microbiota-dependent induction of EMT or stromal reprogramming in CCA remains limited.

Therefore, this pathway is classified as C2 evidence, reflecting a mechanistically coherent model supported by stromal biology and inflammation studies, but without definitive microbiome-controlled validation in CCA.

### 6.6 Immune-checkpoint regulation and antigen-presentation defects

#### 6.6.1 Subtype relevance: Pan-CCA

##### 6.6.1.1 Highest level of evidence: C2

Microbiome-associated inflammation can reshape tumor immune landscapes by promoting myeloid cell expansion, impairing antigen presentation, and upregulating immune checkpoint molecules such as PD-1 and PD-L1 (Greten et al., 2023; Huang et al., 2025). These processes collectively weaken cytotoxic T-cell responses and may contribute to immune evasion in CCA.

In CCA, observational studies have reported associations between dysbiosis, altered immune infiltration patterns, and heterogeneous responses to immune checkpoint blockade (Fu et al., 2025). Broader cancer immunology research provides strong evidence that microbial composition influences immune checkpoint signaling and immunotherapy efficacy (Jia et al., 2024; Gazzaniga and Kasper, 2025).

However, direct mechanistic studies linking specific microbial taxa or metabolites to immune checkpoint regulation in CCA are currently scarce, and most functional insights are extrapolated from other tumor types.

Accordingly, this mechanism is assigned a C2 evidence level, reflecting indirect but biologically consistent support derived from immune intervention and translational studies, with limited CCA-specific mechanistic validation.

### 6.7 Block III. Emerging and hypothesis-generating mechanisms (C0–C2)

#### 6.7.1 Microbial genotoxins and direct DNA damage

##### 6.7.1.1 Subtype relevance: parasite-associated CCA, pCCA

###### 6.7.1.1.1 Highest level of evidence: C1–C2

Certain bacteria produce genotoxins, such as colibactin and cytolethal distending toxin, which induce DNA cross-links, double-strand breaks, and characteristic mutational signatures. Colibactin-associated mutational footprints have been identified in multiple human cancers, supporting a plausible route by which enteric or biliary bacteria might initiate or accelerate mutagenesis in biliary epithelium (He et al., 2019; Dziubańska-Kusibab et al., 2020; Berger and Meyer, 2021).

In parasite-associated CCA, carcinogenesis may be driven by microbe-mediated production of carcinogenic metabolites rather than direct genotoxicity alone. *Opisthorchis viverrini* infection appears to facilitate microbial translocation into the bile duct, reshaping biliary metabolic output and promoting accumulation of pro-tumorigenic metabolites such as bile acids and ammonia, thereby creating a permissive microenvironment for malignant transformation (Chng et al., 2016).

However, direct demonstration of microbial genotoxin activity within CCA tissue is lacking, and most evidence remains associative or inferred from other malignancies and infection models.

Thus, this mechanism is classified as C1–C2 evidence, reflecting biologically plausible, partially supported hypotheses that require direct validation in CCA-specific systems.

### 6.8 Microbial metabolites and epigenetic remodeling

#### 6.8.1 Subtype relevance: pan-CCA (hypothesized)

##### 6.8.1.1 Highest level of evidence: C0–C1

Emerging evidence suggests that microbial metabolites can influence host epigenetic landscapes, including DNA methylation, histone modifications, and chromatin accessibility, thereby reshaping cancer-related transcriptional programs. Short-chain fatty acids, particularly butyrate, have been shown to function as histone deacetylase inhibitors, modulating gene expression programs involved in cell differentiation, immune regulation, and tumor suppression in colorectal and gastric cancers (Mirzaei et al., 2021; Wang et al., 2024; Liu et al., 2025).

In the context of cholangiocarcinoma, direct experimental evidence linking microbiota-derived metabolites to epigenetic remodeling remains limited. However, biliary epithelial cells are continuously exposed to bile acids and microbial products originating from the gut–liver axis, suggesting biological plausibility for epigenetic modulation in CCA. Supporting evidence from hepatocellular carcinoma and colorectal cancer demonstrates that microbial dysbiosis can reshape host epigenetic states through altered metabolite availability and inflammatory signaling, indirectly implicating similar mechanisms in CCA (Qu et al., 2023; Yoon et al., 2025).

Importantly, the extrapolation of these findings to CCA involves multiple inferential steps, including differences in epithelial lineage, microenvironmental exposure, and immune contexture. Therefore, while microbiota-driven epigenetic remodeling represents a conceptually compelling mechanism, its role in CCA currently rests on indirect evidence and cross-cancer extrapolation rather than direct validation.

Accordingly, this mechanism is classified as C0–C1 level evidence, reflecting a hypothesis-generating framework supported by mechanistic studies in related gastrointestinal malignancies, but lacking direct experimental confirmation in cholangiocarcinoma models.

### 6.9 Phage–bacteria dynamics: an emerging ecological layer

#### 6.9.1 Subtype relevance: pan-CCA

##### 6.9.1.1 Highest level of evidence: C0

Bacteriophages shape bacterial community structure and selectively regulate microbial functional capacity, thereby indirectly influencing carcinogenic pathways, including selection for genotoxin-producing or antibiotic-resistant strains. Although phage–bacteria dynamics have been shown to influence tumor biology in other contexts, evidence in CCA is currently limited to ecological inference (Manivannan et al., 2022).

No direct studies have examined phage composition or phage-mediated functional shifts in CCA bile or tissue samples. Consequently, proposed roles for phage-based modulation in CCA remain speculative but conceptually attractive, particularly as a future therapeutic avenue.

Therefore, this mechanism is classified as C0 evidence, representing an exploratory, hypothesis-generating concept lacking direct empirical support in CCA.

### 6.10 Integration across subtypes and translational implications

Mechanistic relevance varies by subtype: iCCA is particularly linked to bile-acid signaling, metabolic inflammation, and ferroptosis regulation (Jia et al., 2020; Chai et al., 2023; Zhang et al., 2024); pCCA/dCCA mechanisms are often dominated by obstruction-related biofilm formation, ERCP/stent-introduced microbes, and reflux-mediated epithelial injury (Chen et al., 2019; Kayashima et al., 2025; Meacci et al., 2025); fluke-associated CCA features parasite–microbiome synergy, nitrosamine production, and sustained genotoxic inflammation (Chng et al., 2016; Chen et al., 2023). These subtype distinctions imply that mechanistic hypotheses from one CCA category cannot be generalized without validation in subtype-matched cohorts.

The available evidence has now progressed beyond isolated associative observations, revealing convergent mechanistic signals across multiple biological axes, including metabolic signaling, microbial genotoxicity, immune remodeling, epigenetic reprogramming, and ecological selection within the tumor microenvironment. Importantly, these processes are unlikely to operate as independent or linear pathways. Instead, they are best viewed as interconnected and mutually reinforcing mechanisms, whereby metabolic alterations shape immune states, immune pressure influences microbial ecology, and microbial metabolites and inflammatory cues jointly remodel epithelial and stromal programs.

Nevertheless, despite this emerging systems-level framework, most mechanistic links remain provisional and context-dependent, with limited direct causal validation in cholangiocarcinoma. Rigorous confirmation will require integrative spatial multi-omics, controlled microbial perturbation approaches (including gnotobiotic and organoid-based models), and prospective interventional studies that explicitly account for cholangiocarcinoma subtype heterogeneity.

## 7 Microbiome-targeted therapeutic strategies for CCA

**Figure 5:**
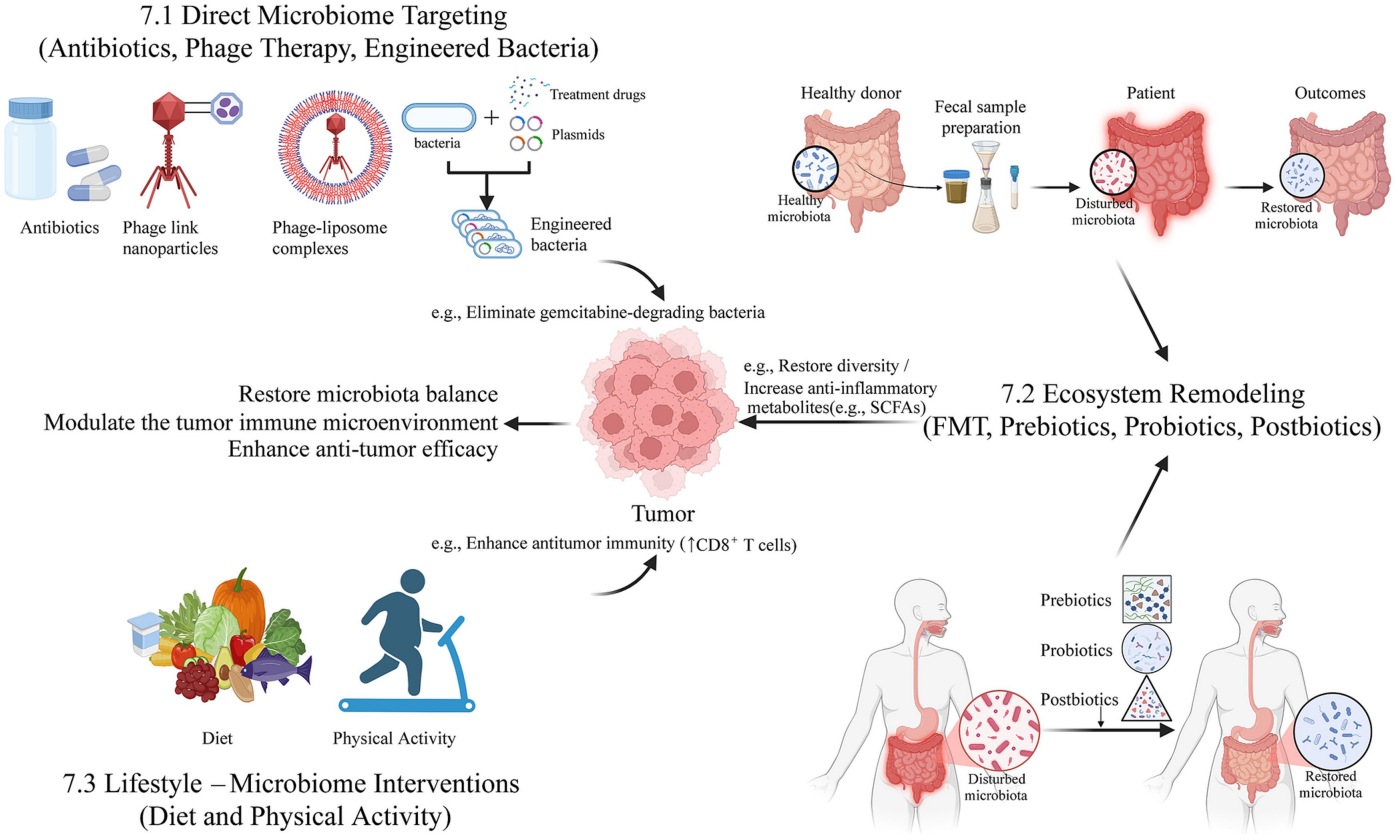
The figure illustrates three principal clinical strategies derived from CCA microbiome research: Direct Antimicrobial Targeting, Microbiome Remodeling, and Lifestyle–Microbiome Interventions. Strategies focus on eliminating specific bacteria (e.g., *gamma-Proteobacteria*) to sensitize chemotherapy, and leveraging FMT or defined probiotics to enhance the efficacy of Immune Checkpoint Inhibitors. Leveraging exercise or dietary regulation to increase beneficial microbial metabolites (e.g., Formate), which indirectly promotes the anti-tumor activity of CD8^+^T cells.

Recent studies highlight the microbiome as a modifiable determinant of CCA progression and treatment response. Accordingly, microbiome-based interventions can be conceptualized into three major categories: direct microbial targeting, ecosystem remodeling, and lifestyle-driven microbial modulation (Figure 5). Microbiome science offers a promising avenue for developing novel, targeted adjuvant therapies aimed at improving outcomes and enhancing treatment response by modulating the microbial environment (Wu et al., 2025).

### 7.1 Direct microbiome targeting (antibiotics, phage therapy, engineered bacteria)

Direct targeting strategies aim to eliminate or suppress oncogenic microbiota and interrupt microbe-driven tumor pathways. Broad-spectrum or selective antibiotics, such as neomycin, have shown efficacy in reducing microbial load and blocking the LPS–TLR4 axis, thereby limiting the recruitment of immunosuppressive PMN-MDSCs and restraining tumor progression (Zhang et al., 2021; Patangia et al., 2022). Highly specific approaches—including bacteriophage therapy and engineered bacterial strains—offer the possibility of selectively eradicating CCA-associated taxa such as *gamma-Proteobacteria*, which have been implicated in chemotherapy resistance (Ito et al., 2022; Shuwen and Kefeng, 2022; Ahmed et al., 2025). Although these targeted modalities are still largely theoretical in CCA, they represent promising candidates for precision microbial depletion strategies with fewer off-target effects than conventional antibiotics.

### 7.2 Ecosystem remodeling (FMT, probiotics, prebiotics)

Ecosystem remodeling focuses on restoring microbial diversity and beneficial functions rather than eliminating specific taxa. FMT and supplementation with probiotics or prebiotics aim to replenish beneficial genera (e.g., *Bifidobacterium*, *Lactobacillus*) and increase short-chain fatty acids, which exert anti-inflammatory and anti-tumor effects (Chen et al., 2019; Davar et al., 2021; Zhang et al., 2023; Ye et al., 2025). Although research in CCA remains preliminary, FMT holds potential for reducing pro-carcinogenic metabolites and reshaping the immune microenvironment. Early data from other cancer types suggest that microbiome restoration may enhance responses to immunotherapies, providing a strong rationale for evaluating these interventions in CCA (Lin et al., 2025).

### 7.3 Lifestyle–microbiome interventions (diet and physical activity)

A highly feasible, non-pharmacological extension of ecosystem remodeling involves Dietary and Lifestyle Modifications. These factors are master regulators of gut microbial structure and metabolic output, influencing tumor progression in various cancers (Saha et al., 2024). Dietary interventions that increase microbial diversity or SCFA production may help counteract microbe-driven inflammation and tumor growth (Singh et al., 2018). Emerging evidence also indicates that physical exercise modulates microbial metabolites capable of enhancing CD8^+^T cell–mediated anti-tumor immunity, thereby improving immunotherapy efficacy (Phelps et al., 2025). Because these interventions are safe, inexpensive, and non-pharmacological, optimizing diet and exercise represents a feasible strategy to prime the host immune system before systemic therapy in CCA.

### 7.4 Microbiome-driven treatment modification (chemotherapy resistance and immunotherapy response)

The microbiome acts as a potent modifier of treatment efficacy. A well-established example is the finding that *gamma-Proteobacteria* can express long-form cytidine deaminase, enzymatically inactivating gemcitabine and promoting chemotherapy resistance. Targeting such taxa or inhibiting microbial drug-metabolizing enzymes may enhance chemotherapeutic potency (Sitthirak et al., 2022). Microbial influences on immunotherapy are also increasingly recognized; dysbiosis-driven inflammation, myeloid expansion, and altered metabolite profiles can suppress anti-tumor immunity and reduce responsiveness to immune checkpoint inhibitors (Zhou et al., 2021; Jiang et al., 2023). Therapeutically manipulating these pathways offers a rational strategy to sensitize CCA tumors to immunotherapy.

### 7.5 Limitations and future clinical trial priorities

Despite their promise, most microbiome-based interventions for CCA remain in early experimental phases and lack validation in large, well-controlled clinical trials. Future studies must evaluate safety, durability, and mechanistic specificity using randomized controlled trial designs, ideally incorporating multi-omics profiling to identify predictive microbial signatures. Integrating microbial modulation with standard chemotherapy, immunotherapy, and targeted therapy is likely to yield the most significant clinical advances, paving the way toward personalized, microbiome-informed management of CCA.

### 7.6 Clinical translation barriers and practical considerations

Despite growing mechanistic and preclinical evidence supporting microbiome-targeted interventions in CCA, several substantial barriers currently limit their clinical implementation. These challenges span evidence maturity, disease-specific safety concerns, regulatory complexity, and realistic timelines for translation.

(a) Current clinical trial landscape

To date, most microbiome-based therapeutic strategies—including FMT, defined microbial consortia, phage therapy, and engineered bacteria—have entered clinical testing primarily in non-oncologic indications or in selected cancer types, most notably melanoma and colorectal cancer, often in combination with immune checkpoint inhibitors.

FMT represents the most clinically mature microbiome-based intervention. In oncology, early-phase trials in melanoma demonstrated that FMT from immune checkpoint inhibitor (ICI) responders could partially restore responsiveness to anti–PD-1 therapy in refractory patients. In a phase I trial by Baruch et al., 10 anti–PD-1–refractory melanoma patients received FMT followed by anti–PD-1 rechallenge, resulting in objective responses in 3 patients (30%), with no grade ≥3 FMT-related adverse events reported (Baruch et al., 2021). Similarly, Davar et al. treated 15 patients with PD-1–refractory metastatic melanoma, observing objective responses or durable disease control in 6 patients (40%), with treatment-related toxicities largely limited to grade 1–2 gastrointestinal symptoms and no unexpected infectious complications (Davar et al., 2021). In addition, cohort studies and systematic reviews assessing FMT for recurrent *Clostridioides difficile* infection in cancer patients—including those with advanced solid tumors—have reported clinical cure rates ranging from 80 to 90%, with serious adverse events occurring in approximately 3–5% of cases under stringent donor screening protocols (Ali et al., 2021).

Collectively, these studies indicate that FMT can be administered to carefully selected oncology patients with an acceptable short-term safety profile. However, it is important to emphasize that most available trials are small, non-randomized, and underpowered to evaluate antitumor efficacy, and none were designed to address CCA specifically. To date, no microbiome-targeted intervention has been evaluated in dedicated interventional clinical trials for CCA, and all current evidence in this disease remains extrapolated from other malignancies or derived from preclinical and observational studies, underscoring the early translational stage of this field (Yang et al., 2024; Wekking et al., 2025).

(b) Safety concerns specific to CCA patients

Patients with CCA present unique safety vulnerabilities that substantially complicate microbiome-based interventions. Biliary obstruction, prior ERCP, indwelling biliary stents, and recurrent cholangitis increase the baseline risk of bacteremia and sepsis (Qurashi et al., 2025). These risks are further amplified in patients receiving chemotherapy or immune checkpoint inhibitors.

In this context, FMT raises specific concerns related to donor selection, microbial translocation, and infectious complications. Regulatory agencies have highlighted these risks following reports of fatal bacteremia caused by multidrug-resistant organisms transmitted via FMT in immunocompromised recipients, prompting stricter donor screening and safety oversight (DeFilipp et al., 2019; Song et al., 2025). For CCA patients with disrupted biliary and intestinal barriers, the risk of systemic dissemination of transplanted microbes represents a nontrivial concern. Optimal timing of FMT relative to surgery, biliary drainage, antibiotic exposure, or systemic therapy also remains undefined.

Similarly, phage therapy and engineered bacterial approaches pose additional biosafety and regulatory challenges, including off-target ecological effects, horizontal gene transfer, manufacturing standardization, and long-term monitoring requirements (Ting et al., 2022; Blake et al., 2024; Abd El-Moaty et al., 2025). These issues are especially pertinent in oncology patients with compromised mucosal barriers and altered immune surveillance, and have been consistently emphasized as central limitations in the broader microbiome therapeutics literature.

(c) Realistic timelines and translational outlook

Taken together, microbiome-targeted therapies in CCA should currently be regarded as medium- to long-term translational goals rather than near-term clinical solutions. From a regulatory and developmental perspective, different classes of microbiome-based interventions are positioned at distinct stages of clinical translation and therefore follow different anticipated timelines.

In the short term, the most feasible applications are likely indirect microbiome-informed strategies, such as patient stratification, biomarker development, antibiotic stewardship, or modulation of treatment response, rather than direct microbial replacement or engineering. Conventional small-molecule antibiotics or microbiome-modulating drugs that do not involve live organisms may follow more traditional regulatory pathways, with estimated development timelines of approximately 5–7 years from IND initiation to potential approval, if targeted toward microbiome-related endpoints (Thanush and Venkatesh, 2023; Cha and Sonu, 2025).

By contrast, FMT, when conducted under an Investigational New Drug (IND) framework as required by regulatory agencies such as the U. S. Food and Drug Administration, faces additional requirements for donor screening, safety monitoring, and disease-specific efficacy demonstration. For novel oncologic indications beyond recurrent *Clostridioides difficile* infection, IND-driven Phase I/II trials in CCA would likely require 3–5 years to generate preliminary safety and efficacy data, with 5–10 years or longer needed to progress toward Phase III trials and potential regulatory submission, reflecting typical development timelines for complex biological products (Thanush and Venkatesh, 2023; Cha and Sonu, 2025).

Next,-generation microbiome therapeutics—including defined live biotherapeutic products, genetically engineered bacterial strains, and phage therapies—remain at even earlier developmental stages. These approaches face substantial regulatory and manufacturing challenges, including preclinical optimization, biosafety characterization, and Good Manufacturing Practice (GMP) scale-up prior to first-in-human testing. Based on current regulatory frameworks for live biologics, engineered bacterial and phage-based therapies are realistically positioned for initial Phase I evaluation in oncology settings within the next 5–8 years, with Phase II proof-of-concept studies potentially extending into the subsequent 5-year period, contingent on favorable safety profiles and sustained regulatory engagement.

To further contextualize the translational maturity of microbiome-targeted strategies in cholangiocarcinoma, we adapted a Technology Readiness Level (TRL) framework to systematically compare major intervention classes, including antibiotics, FMT, phage therapy, and engineered live biotherapeutic products (LBPs). This framework integrates current clinical evidence, regulatory status, manufacturing feasibility, and safety considerations, providing a comparative overview of readiness for clinical translation specifically in the CCA setting (Table 9).

**Table 9:** Intervention readiness levels for microbiome-targeted strategies in the context of CCA.

Intervention	Current clinical stage	Representative evidence	Estimated TRL	Key translational barriers
Antibiotics/antibiotic stewardship	Routine clinical use	Broad use in cancer patients with infection; observational links with outcome	TRL 8–9	Off-target microbiome effects; resistance; confounding clinical variables
Fecal Microbiota Transplantation (FMT)	Early exploratory oncology trials (e.g., melanoma, hematologic GVHD)	Phase I melanoma FMT + PD-1 (n = 10–15, ORR ~ 30–40%, low grade AE)	TRL 5–6	Infection risk, donor screening, regulatory IND requirements; absence of CCA-specific trials
Phage therapy	Preclinical / Phase I in limited indications	Early phage clinical trials in non-CCA infections; no oncology phage RCTs	TRL 3–4	Target specificity, delivery, regulatory classification, safety
Engineered bacteria (LBPs)	Preclinical / early Phase I in other contexts	Engineered bacterial therapeutics in safety/tumor models (non-CCA)	TRL 2–3	Biosafety, manufacturing standardization, host control, regulatory classification

Progress toward clinical translation across all microbiome-based modalities will require carefully designed early-phase clinical trials incorporating stringent safety monitoring, rigorous donor screening (where applicable), standardized manufacturing pipelines, and subtype-specific enrollment strategies. Such studies will be essential to establish safety, durability, and mechanistic specificity before microbiome-targeted interventions can be responsibly advanced toward regulatory approval and clinical adoption in CCA.

## 8 Discussion

Microbiome research in CCA has progressed from preliminary association studies to functional validation of specific tumor-promoting pathways, underscoring the microbiome as a critical driver of CCA initiation, progression, and therapeutic resistance. Key mechanisms identified to date include gut microbial translocation activating the TLR4–CXCL1–MDSC immunosuppressive axis (Zhang et al., 2021), *Helicobacter pylori*–induced fibro-inflammatory remodeling (Klungsaeng et al., 2025), and *gamma-Proteobacteria*–mediated gemcitabine inactivation (Sitthirak et al., 2022). Together, these studies provide biologically plausible links between microbial dysbiosis and malignant behavior in CCA.

Despite these advances, interpretation of the existing literature is constrained by several methodological limitations. Most human CCA microbiome studies are based on relatively small cohorts and are vulnerable to clinical confounders, including biliary obstruction, cholangitis, antibiotic exposure, and prior endoscopic interventions (see Section 3.1). In addition, the low microbial biomass of bile and tumor tissues increases susceptibility to environmental and reagent contamination, necessitating rigorous controls and contamination-aware analytical pipelines (see Section 3.2). Diagnostic models reporting near-perfect AUC values should therefore be interpreted cautiously, as overfitting and cohort-specific effects cannot be excluded (see Section 5). These factors are likely to generate spurious or context-dependent microbiome signatures and may partially explain inconsistencies across reported CCA cohorts.

Beyond these study-level limitations, an additional challenge lies in the substantial heterogeneity in methodological rigor across the existing literature. Across the current literature, substantial methodological heterogeneity limits the extent to which microbiome–CCA associations can be meta-interpreted or generalized. Studies vary widely in sample type (fecal, bile, tissue), sequencing platforms, bioinformatic pipelines, confounder adjustment, and cohort composition, with many relying on small, single-center, cross-sectional designs. A significant proportion of available evidence derives from exploratory or moderate-quality studies. This heterogeneity increases the risk of overinterpretation and may partially explain inconsistencies across reported microbial signatures. Accordingly, associations highlighted in this review should be interpreted within the context of their methodological rigor, and future progress will depend on standardized study designs, rigorous contamination control, and prospective validation in well-phenotyped, multi-center cohorts.

A particularly important unresolved issue is reverse causality. The majority of available studies are cross-sectional (C1-level) and are conducted at the time of diagnosis, when most patients already present with advanced disease and varying degrees of biliary obstruction. As a result, these studies cannot establish temporal precedence, and it remains unclear whether observed microbial alterations represent causal drivers of cholangiocarcinogenesis or secondary consequences of tumor growth, impaired bile flow, inflammation, and clinical management. Biliary obstruction alters bile acid composition and antimicrobial pressure, while recurrent cholangitis, systemic antibiotics, ERCP, and biliary stenting can profoundly reshape local and gut microbial communities or introduce exogenous microbes and biofilms (Qurashi et al., 2025). These factors are likely to generate spurious or context-dependent microbiome signatures and may partially explain inconsistencies across reported CCA cohorts.

Addressing causality will require study designs that extend beyond cross-sectional sampling. Priority approaches include prospective longitudinal cohorts in high-risk populations such as patients with primary sclerosing cholangitis, pre-diagnostic biobanking with serial microbiome and bile acid profiling, and careful integration of clinical metadata capturing obstruction status, procedures, and antimicrobial exposure. In parallel, interventional studies—such as microbiome-modulating therapies or antibiotic de-escalation strategies with predefined microbial, metabolic, and immunologic endpoints—will be essential to determine whether altering specific microbial pathways can modify CCA risk or treatment response. Collectively, these efforts are necessary to move the field from correlation toward causation and to translate microbiome insights into clinically actionable strategies for CCA prevention, diagnosis, and therapy.

## 9 Future perspectives: actionable research road map

The future trajectory of CCA microbiome research must shift from purely descriptive association to actionable, hypothesis-driven translation, necessitating focus on three key priorities.

### 9.1 Causal and high-resolution mechanistic validation

Breaking the bottleneck of observational association is the primary objective of future research. This requires leveraging C4 level studies that utilize Germ-Free (GF) or specific genetic animal models (such as CCA patient-derived xenograft, PDX) to perform causal testing by colonizing or transplanting CCA-specific dysbiotic microbiota (e.g., *Klebsiella or Fusobacterium*) to verify their capacity to accelerate CCA onset and progression (Miyabe et al., 2022; Ye et al., 2025). Simultaneously, spatial and functional omics validation is essential: this includes integrating spatial transcriptomics or single-cell RNA sequencing with FISH to accurately map the spatial distribution of microbial communities within tumor tissue. Such high-resolution analyses will determine if specific strains are functionally active *in situ* (via meta-transcriptomics/meta-proteomics) and define their focal interaction with host cells (e.g., MDSCs, CAFs) (Zhang et al., 2021; Klungsaeng et al., 2025). Ultimately, multi-omics integration—combining bacterial, fungal, and metabolomics data—is needed to construct dynamic regulatory networks of the microbe-metabolite-host signal, aiming to identify and quantify functionally active carcinogenic or cancer-suppressing metabolites.

### 9.2 Clinical translation and intervention trials

The core goal of translational research is to advance microbial biomarkers and intervention strategies to clinical validation. This demands the immediate launch of large-scale, multi-center, prospective longitudinal cohorts that collect samples at multiple time points (pre-diagnosis, mid-treatment, post-relapse). A critical procedural requirement for these studies is the paired collection of a patient’s stool (gut), bile, and tumor tissue to accurately track the microbial translocation axis from the gut to the biliary system and tumor (Isali et al., 2024). Furthermore, currently reported high AUC diagnostic models must undergo rigorous independent external validation in separate cohorts to ensure clinical generalizability and stability (Li et al., 2023). The most impactful task involves conducting randomized controlled trials (RCTs): for chemotherapy sensitization, trials should test targeted interventions (e.g., phage therapy or selective antibiotics) designed to eliminate intratumoral *gamma-Proteobacteria*, using objective response rate to Gemcitabine/Cisplatin as the primary clinical endpoint; for immunotherapy synergy, RCTs involving FMT or probiotic interventions should be conducted in CCA patients to assess the enhanced efficacy of PD-1/PD-L1 inhibitors.

### 9.3 Methodological standardization priorities

To ensure the reliability and comparability of research data, methodological standardization must be prioritized. Study designs must rigorously document and strictly exclude key clinical confounders, including biliary obstruction, stent placement, and recent antibiotic use history. Quality control is particularly essential for low-biomass samples (bile and tissue), where studies should mandate the inclusion of both extraction and PCR negative controls and employ methods such as qPCR to quantify the absolute bacterial load, thereby distinguishing true microbial signals from sequencing artifacts (Selway et al., 2020; Fierer et al., 2025). By implementing this specific, actionable research roadmap, CCA microbiome research is poised to transition from a highly relevant exploratory field into a clinical driver for developing early diagnostic biomarkers and novel auxiliary therapeutic strategies.

## Glossary

CCA: Cholangiocarcinoma
iCCA/ICC: Intrahepatic CCA
pCCA: Perihilar CCA
dCCA: Distal CCA
GUDCA: Glycoursodeoxycholic acid
TUDCA: Tauroursodeoxycholic acid
PSC: Primary sclerosing cholangitis
PMN-MDSCs: Polymorphonuclear myeloid-derived suppressor cells
LC: Liver Cirrhosis
HCC: Hepatocellular carcinoma
CH: Cholelithiasis
HC: Healthy control
BTC: Biliary tract cancer
BBD: Benign biliary disease
GWAS: Genome-wide association studies
PC: Pancreatic cancer
eCCA: Extrahepatic CCA
BBP: Benign biliary pathology
Ova: *Opisthorchis viverrine*
PT: Paracancerous tissue
BDL: Bile duct ligation
LPS: lipopolysaccharide
ROC: Receiver Operating Characteristic curve
AUC: Area under the ROC Curve
MR: Mendelian Randomization
TME: Tumor microenvironment
ERCP: Endoscopic retrograde cholangiopancreatography
CA199: Carbohydrate antigen199
OS: Overall survival
RFS: Recurrence-free survival
PFS: Progression-free survival
EVs: Extracellular vesicles
*H. pylori*: *Helicobacter pylori*
CAFs: Cancer-associated fibroblasts
CBR: Clinical benefit group
NCB: Non-clinical benefit group
TEM: Transmission electron microscopy
FISH: Fluorescence in situ Hybridization
NGS: Next-generation sequencing
FMT: Fecal microbiome transplantation

## References

[ref1] Abd El-Moaty H. I. Doghish A. S. Moustafa H. A. M. Elkalla W. S. Sayed G. A. Elshami N. H. . (2025). Phage therapy and its role in cancer treatment and control. Folia Microbiol. (Praha) 70, 941–960. doi: 10.1007/s12223-025-01342-9, 40963058

[ref2] Abed J. Emgård J. E. Zamir G. Faroja M. Almogy G. Grenov A. . (2016). Fap2 mediates *Fusobacterium nucleatum* colorectal adenocarcinoma enrichment by binding to tumor-expressed Gal-GalNAc. Cell Host Microbe 20, 215–225. doi: 10.1016/j.chom.2016.07.006, 27512904 PMC5465824

[ref3] Adam P. S. Bornemann T. L. V. Probst A. J. (2022). Progress and challenges in studying the ecophysiology of Archaea. Methods Mol. Biol. 2522, 469–486. doi: 10.1007/978-1-0716-2445-6_32, 36125771

[ref4] Aggarwal N. Kitano S. Puah G. R. Y. Kittelmann S. Hwang I. Y. Chang M. W. (2023). Microbiome and human health: current understanding, engineering, and enabling technologies. Chem. Rev. 123, 31–72. doi: 10.1021/acs.chemrev.2c00431, 36317983 PMC9837825

[ref5] Agudelo J. Miller A. W. (2025). Impact of study design, contamination, and data characteristics on results and interpretation of microbiome studies. mSystems 10:e0040825. doi: 10.1128/msystems.00408-25, 40767516 PMC12456016

[ref6] Ahmed T. Xu X. Noman M. Wang Q. Li B. (2025). Phage-guided nanocarriers: a precision strategy against bacterial pathogens. Trends Biotechnol. 43, 494–497. doi: 10.1016/j.tibtech.2024.09.002, 39341741

[ref7] Ali H. Khurana S. Ma W. Peng Y. Jiang Z. D. DuPont H. . (2021). Safety and efficacy of fecal microbiota transplantation to treat and prevent recurrent *Clostridioides difficile* in cancer patients. J. Cancer 12, 6498–6506. doi: 10.7150/jca.59251, 34659541 PMC8489149

[ref8] Al-Kabban A. Al-Kabban F. M. Obaid O. (2025). Post-endoscopic retrograde cholangiopancreatography (ERCP) complications: A systematic review of microbial patterns, incidence, risk factors, and management strategies in contemporary practice. Cureus 17:e88043. doi: 10.7759/cureus.88043, 40821288 PMC12357005

[ref9] Altveş S. Yildiz H. K. Vural H. C. (2020). Interaction of the microbiota with the human body in health and diseases. Biosci. Microbiota Food Health 39, 23–32. doi: 10.12938/bmfh.19-023, 32328397 PMC7162693

[ref10] An J. Kim D. Oh B. Oh Y. J. Song J. Park N. . (2022). Comprehensive characterization of viral integrations and genomic aberrations in HBV-infected intrahepatic cholangiocarcinomas. Hepatology 75, 997–1011. doi: 10.1002/hep.32135, 34478159

[ref11] Armengaud J. (2025). The dawn of the revolution that will allow us to precisely describe how microbiomes function. J. Proteome 316:105430. doi: 10.1016/j.jprot.2025.105430, 40081757

[ref12] Auslander N. Gussow A. B. Benler S. Wolf Y. I. Koonin E. V. (2020). Seeker: alignment-free identification of bacteriophage genomes by deep learning. Nucleic Acids Res. 48:e121. doi: 10.1093/nar/gkaa856, 33045744 PMC7708075

[ref13] Avershina E. Qureshi A. I. Winther-Larsen H. C. Rounge T. B. (2025). Challenges in capturing the mycobiome from shotgun metagenome data: lack of software and databases. Microbiome 13:66. doi: 10.1186/s40168-025-02048-3, 40055808 PMC11887097

[ref14] Avilés-Jiménez F. Guitron A. Segura-López F. Méndez-Tenorio A. Iwai S. Hernández-Guerrero A. . (2016). Microbiota studies in the bile duct strongly suggest a role for *Helicobacter pylori* in extrahepatic cholangiocarcinoma. Clin. Microbiol. Infect. 22, 178.e11–78.e22. doi: 10.1016/j.cmi.2015.10.008, 26493848

[ref15] Aykut B. Pushalkar S. Chen R. Li Q. Abengozar R. Kim J. I. . (2019). The fungal mycobiome promotes pancreatic oncogenesis via activation of MBL. Nature 574, 264–267. doi: 10.1038/s41586-019-1608-2, 31578522 PMC6858566

[ref16] Bäckhed F. Ley R. E. Sonnenburg J. L. Peterson D. A. Gordon J. I. (2005). Host-bacterial mutualism in the human intestine. Science 307, 1915–1920. doi: 10.1126/science.1104816, 15790844

[ref17] Baruch E. N. Youngster I. Ben-Betzalel G. Ortenberg R. Lahat A. Katz L. . (2021). Fecal microbiota transplant promotes response in immunotherapy-refractory melanoma patients. Science 371, 602–609. doi: 10.1126/science.abb5920, 33303685

[ref18] Berger H. Meyer T. F. (2021). Mechanistic dissection unmasks colibactin as a prevalent mutagenic driver of cancer. Cancer Cell 39, 1439–1441. doi: 10.1016/j.ccell.2021.10.010, 34752751

[ref19] Bertocchi A. Carloni S. Ravenda P. S. Bertalot G. Spadoni I. Lo Cascio A. . (2021). Gut vascular barrier impairment leads to intestinal bacteria dissemination and colorectal cancer metastasis to liver. Cancer Cell 39, 708–24.e11. doi: 10.1016/j.ccell.2021.03.004, 33798472

[ref20] Bhatt A. P. Redinbo M. R. Bultman S. J. (2017). The role of the microbiome in cancer development and therapy. CA Cancer J. Clin. 67, 326–344. doi: 10.3322/caac.21398, 28481406 PMC5530583

[ref21] Blake S. J. Wolf Y. Boursi B. Lynn D. J. (2024). Role of the microbiota in response to and recovery from cancer therapy. Nat. Rev. Immunol. 24, 308–325. doi: 10.1038/s41577-023-00951-0, 37932511

[ref22] Bokulich N. A. Ziemski M. Robeson M. S. Kaehler B. D. (2020). Measuring the microbiome: best practices for developing and benchmarking microbiomics methods. Comput. Struct. Biotechnol. J. 18, 4048–4062. doi: 10.1016/j.csbj.2020.11.049, 33363701 PMC7744638

[ref23] Boonyanugomol W. Chomvarin C. Sripa B. Bhudhisawasdi V. Khuntikeo N. Hahnvajanawong C. . (2012). *Helicobacter pylori* in Thai patients with cholangiocarcinoma and its association with biliary inflammation and proliferation. HPB (Oxford) 14, 177–184. doi: 10.1111/j.1477-2574.2011.00423.x, 22321036 PMC3371200

[ref24] Camille E. Sébastien B. Virginie B. (2026). The hidden players: the mycobiome of pancreatic ductal adenocarcinoma tumors. Microbiol. Res. 303:128392. doi: 10.1016/j.micres.2025.128392 41205302

[ref25] Cha R. R. Sonu I. (2025). Fecal microbiota transplantation: present and future. Clin. Endosc. 58, 352–359. doi: 10.5946/ce.2024.270, 40468650 PMC12138360

[ref26] Chai X. Wang J. Li H. Gao C. Li S. Wei C. . (2023). Intratumor microbiome features reveal antitumor potentials of intrahepatic cholangiocarcinoma. Gut Microbes 15:2156255. doi: 10.1080/19490976.2022.2156255, 36563106 PMC9794006

[ref27] Chaudhary P. P. Conway P. L. Schlundt J. (2018). Methanogens in humans: potentially beneficial or harmful for health. Appl. Microbiol. Biotechnol. 102, 3095–3104. doi: 10.1007/s00253-018-8871-2, 29497795

[ref28] Chen B. Fu S. W. Lu L. Zhao H. (2019). A preliminary study of biliary microbiota in patients with bile duct stones or distal cholangiocarcinoma. Biomed. Res. Int. 2019:1092563. doi: 10.1155/2019/1092563, 31662965 PMC6778921

[ref29] Chen R. Li X. Ding J. Wan J. Zhang X. Jiang X. . (2023). Profiles of biliary microbiota in biliary obstruction patients with *Clonorchis sinensis* infection. Front. Cell. Infect. Microbiol. 13:1281745. doi: 10.3389/fcimb.2023.1281745, 38164415 PMC10757933

[ref30] Chen B. Liu X. Yu P. Xie F. Kwan J. S. H. Chan W. N. . (2023). H. pylori-induced NF-κB-PIEZO1-YAP1-CTGF axis drives gastric cancer progression and cancer-associated fibroblast-mediated tumour microenvironment remodelling. Clin. Transl. Med. 13:e1481. doi: 10.1002/ctm2.1481, 37983931 PMC10659770

[ref31] Chen Z. Shi W. Chen K. Lu C. Li X. Li Q. (2023). Elucidating the causal association between gut microbiota and intrahepatic cholangiocarcinoma through Mendelian randomization analysis. Front. Microbiol. 14:1288525. doi: 10.3389/fmicb.2023.1288525, 38033576 PMC10682188

[ref32] Chen D. Wu J. Jin D. Wang B. Cao H. (2019). Fecal microbiota transplantation in cancer management: current status and perspectives. Int. J. Cancer 145, 2021–2031. doi: 10.1002/ijc.32003, 30458058 PMC6767494

[ref33] Chiang J. Y. L. Ferrell J. M. (2018). Bile acid metabolism in liver pathobiology. Gene Expr. 18, 71–87. doi: 10.3727/105221618x15156018385515, 29325602 PMC5954621

[ref34] Chng K. R. Chan S. H. Ng A. H. Q. Li C. Jusakul A. Bertrand D. . (2016). Tissue microbiome profiling identifies an enrichment of specific enteric Bacteria in *Opisthorchis viverrini* associated cholangiocarcinoma. EBioMedicine 8, 195–202. doi: 10.1016/j.ebiom.2016.04.034, 27428430 PMC4919562

[ref35] Choy A. T. F. Carnevale I. Coppola S. Meijer L. L. Kazemier G. Zaura E. . (2018). The microbiome of pancreatic cancer: from molecular diagnostics to new therapeutic approaches to overcome chemoresistance caused by metabolic inactivation of gemcitabine. Expert. Rev. Mol. Diagn. 18, 1005–1009. doi: 10.1080/14737159.2018.1544495, 30392417

[ref36] Clay S. L. Fonseca-Pereira D. Garrett W. S. (2022). Colorectal cancer: the facts in the case of the microbiota. J. Clin. Invest. 132. doi: 10.1172/jci155101, 35166235 PMC8843708

[ref37] Davar D. Dzutsev A. K. McCulloch J. A. Rodrigues R. R. Chauvin J. M. Morrison R. M. . (2021). Fecal microbiota transplant overcomes resistance to anti-PD-1 therapy in melanoma patients. Science 371, 595–602. doi: 10.1126/science.abf3363, 33542131 PMC8097968

[ref38] Davidson R. M. Epperson L. E. (2018). Microbiome sequencing methods for studying human diseases. Methods Mol. Biol. 1706, 77–90. doi: 10.1007/978-1-4939-7471-9_5, 29423794

[ref39] DeFilipp Z. Bloom P. P. Torres Soto M. Mansour M. K. Sater M. R. A. Huntley M. H. . (2019). Drug-Resistant *E. coli* bacteremia transmitted by fecal microbiota transplant. N. Engl. J. Med. 381, 2043–2050. doi: 10.1056/NEJMoa1910437, 31665575

[ref40] Dohlman A. B. Arguijo Mendoza D. Ding S. Gao M. Dressman H. Iliev I. D. . (2021). The cancer microbiome atlas: a pan-cancer comparative analysis to distinguish tissue-resident microbiota from contaminants. Cell Host Microbe 29, 281–98.e5. doi: 10.1016/j.chom.2020.12.001, 33382980 PMC7878430

[ref41] Dougherty M. W. Jobin C. (2023). Intestinal bacteria and colorectal cancer: etiology and treatment. Gut Microbes 15:2185028. doi: 10.1080/19490976.2023.2185028, 36927206 PMC10026918

[ref42] Dziubańska-Kusibab P. J. Berger H. Battistini F. Bouwman B. A. M. Iftekhar A. Katainen R. . (2020). Colibactin DNA-damage signature indicates mutational impact in colorectal cancer. Nat. Med. 26, 1063–1069. doi: 10.1038/s41591-020-0908-2, 32483361

[ref43] Eisenhofer R. Minich J. J. Marotz C. Cooper A. Knight R. Weyrich L. S. (2019). Contamination in low microbial biomass microbiome studies: issues and recommendations. Trends Microbiol. 27, 105–117. doi: 10.1016/j.tim.2018.11.003, 30497919

[ref44] Fickert P. Pollheimer M. J. Beuers U. Lackner C. Hirschfield G. Housset C. . (2014). Characterization of animal models for primary sclerosing cholangitis (PSC). J. Hepatol. 60, 1290–1303. doi: 10.1016/j.jhep.2014.02.006, 24560657 PMC4517670

[ref45] Fierer N. Leung P. M. Lappan R. Eisenhofer R. Ricci F. Holland S. I. . (2025). Guidelines for preventing and reporting contamination in low-biomass microbiome studies. Nat. Microbiol. 10, 1570–1580. doi: 10.1038/s41564-025-02035-2, 40542287

[ref46] Fletcher A. A. Kelly M. S. Eckhoff A. M. Allen P. J. (2023). Revisiting the intrinsic mycobiome in pancreatic cancer. Nature 620, E1–e6. doi: 10.1038/s41586-023-06292-1, 37532819 PMC11062486

[ref47] Fu Y. Bonifacio-Mundaca J. Desterke C. Casafont Í. Mata-Garrido J. (2025). Genomic alterations and microbiota crosstalk in hepatic cancers: the gut-liver Axis in tumorigenesis and therapy. Genes (Basel) 16. doi: 10.3390/genes16080920, 40869967 PMC12385877

[ref48] Gazzaniga F. S. Kasper D. L. (2025). The gut microbiome and cancer response to immune checkpoint inhibitors. J. Clin. Invest. 135. doi: 10.1172/jci184321, 39895632 PMC11785914

[ref49] Greten T. F. Schwabe R. Bardeesy N. Ma L. Goyal L. Kelley R. K. . (2023). Immunology and immunotherapy of cholangiocarcinoma. Nat. Rev. Gastroenterol. Hepatol. 20, 349–365. doi: 10.1038/s41575-022-00741-4, 36697706 PMC12468729

[ref50] Griffard-Smith R. Schueddig E. Mahoney D. E. Chalise P. Koestler D. C. Pei D. (2025). micRoclean: an R package for decontaminating low-biomass 16S-rRNA microbiome data. Front. Bioinform. 5:1556361. doi: 10.3389/fbinf.2025.1556361, 40406150 PMC12095030

[ref51] Han J. Wu S. Fan Y. Tian Y. Kong J. (2021). Biliary microbiota in Choledocholithiasis and correlation with duodenal microbiota. Front. Cell. Infect. Microbiol. 11:625589. doi: 10.3389/fcimb.2021.625589, 33996618 PMC8116743

[ref52] Hannigan G. D. Duhaime M. B. Ruffin M. T. t. Koumpouras C. C. Schloss P. D. (2018). Diagnostic potential and interactive dynamics of the colorectal cancer virome. MBio 9:18. doi: 10.1128/mBio.02248-18, 30459201 PMC6247079

[ref53] Harrison J. M. Visser B. C. (2024). Cholangiocarcinoma. Surg. Clin. North Am. 104, 1281–1293. doi: 10.1016/j.suc.2024.04.003, 39448128

[ref54] He Z. Gharaibeh R. Z. Newsome R. C. Pope J. L. Dougherty M. W. Tomkovich S. . (2019). *Campylobacter jejuni* promotes colorectal tumorigenesis through the action of cytolethal distending toxin. Gut 68, 289–300. doi: 10.1136/gutjnl-2018-317200, 30377189 PMC6352414

[ref55] Heintz-Buschart A. Wilmes P. (2018). Human gut microbiome: function matters. Trends Microbiol. 26, 563–574. doi: 10.1016/j.tim.2017.11.002, 29173869

[ref56] Helmink B. A. Khan M. A. W. Hermann A. Gopalakrishnan V. Wargo J. A. (2019). The microbiome, cancer, and cancer therapy. Nat. Med. 25, 377–388. doi: 10.1038/s41591-019-0377-7, 30842679

[ref57] Hov J. R. Karlsen T. H. (2023). The microbiota and the gut-liver axis in primary sclerosing cholangitis. Nat. Rev. Gastroenterol. Hepatol. 20, 135–154. doi: 10.1038/s41575-022-00690-y, 36352157

[ref58] Huang L. Li Y. Zhang C. Jiang A. Zhu L. Mou W. . (2025). Microbiome meets immunotherapy: unlocking the hidden predictors of immune checkpoint inhibitors. NPJ Biofilms Microbiomes 11:180. doi: 10.1038/s41522-025-00819-2, 40897718 PMC12405452

[ref59] Huang F. Liu Z. Song Y. Wang G. Shi A. Chen T. . (2025). Bile acids activate cancer-associated fibroblasts and induce an immunosuppressive microenvironment in cholangiocarcinoma. Cancer Cell 43, 1460–75.e10. doi: 10.1016/j.ccell.2025.05.017, 40578361

[ref60] Isali I. Wong T. R. Tian S. (2024). Best practice guidelines for collecting microbiome samples in research studies. Eur. Urol. Focus 10, 909–913. doi: 10.1016/j.euf.2024.12.007, 39809682

[ref61] Ito Z. Koido S. Kato K. Odamaki T. Horiuchi S. Akasu T. . (2022). Dysbiosis of the fecal and biliary microbiota in biliary tract Cancer. Cancers (Basel) 14. doi: 10.3390/cancers14215379, 36358797 PMC9653963

[ref62] Jia X. Lu S. Zeng Z. Liu Q. Dong Z. Chen Y. . (2020). Characterization of gut microbiota, bile acid metabolism, and cytokines in intrahepatic cholangiocarcinoma. Hepatology 71, 893–906. doi: 10.1002/hep.30852, 31298745

[ref63] Jia D. Wang Q. Qi Y. Jiang Y. He J. Lin Y. . (2024). Microbial metabolite enhances immunotherapy efficacy by modulating T cell stemness in pan-cancer. Cell 187, 1651–65.e21. doi: 10.1016/j.cell.2024.02.022, 38490195

[ref64] Jiang S. S. Xie Y. L. Xiao X. Y. Kang Z. R. Lin X. L. Zhang L. . (2023). *Fusobacterium nucleatum*-derived succinic acid induces tumor resistance to immunotherapy in colorectal cancer. Cell Host Microbe 31, 781–797. doi: 10.1016/j.chom.2023.04.010, 37130518

[ref65] Kayashima A. Fukuhara S. Miyamoto K. Iwasaki E. Kato M. Sujino T. (2025). Biliary stents reshape the bile microbiome in the absence of cholangitis. Endosc. Int. Open 13:a27333468. doi: 10.1055/a-2733-3468, 41246144 PMC12616555

[ref66] Ke X. Jiang S. Wei Q. Sun M. Sun H. Pang M. . (2025). Unveiling the Intratumor microbiome in liver Cancer: current insights and prospective applications. Clin. Mol. Hepatol. 31, 685–705. doi: 10.3350/cmh.2024.1039, 39838826 PMC12260631

[ref67] Khan A. S. Dageforde L. A. (2019). Cholangiocarcinoma. Surg. Clin. North Am. 99, 315–335. doi: 10.1016/j.suc.2018.12.004, 30846037

[ref68] Khan Mirzaei M. Xue J. Costa R. Ru J. Schulz S. Taranu Z. E. . (2021). Challenges of studying the human Virome - relevant emerging technologies. Trends Microbiol. 29, 171–181. doi: 10.1016/j.tim.2020.05.021, 32622559

[ref69] Kim D. Hofstaedter C. E. Zhao C. Mattei L. Tanes C. Clarke E. . (2017). Optimizing methods and dodging pitfalls in microbiome research. Microbiome 5:52. doi: 10.1186/s40168-017-0267-5, 28476139 PMC5420141

[ref70] Klungsaeng S. Hongsrichan N. Chaidee A. Intuyod K. Pinlaor P. Roytrakul S. . (2025). Melatonin attenuates *Helicobacter pylor*i-mediated cholangiocarcinoma-associated fibroblast activation via modulating integrin/FAK signaling pathway. Sci. Rep. 15:15780. doi: 10.1038/s41598-025-99980-z, 40329017 PMC12056007

[ref71] Koneru S. Thiruvadi V. Ramesh M. (2023). Gut microbiome and its clinical implications: exploring the key players in human health. Curr. Opin. Infect. Dis. 36, 353–359. doi: 10.1097/qco.0000000000000958 37593952

[ref72] Kori M. Gov E. Arga K. Y. Sinha R. (2024). Biomarkers from discovery to clinical application: in silico pre-clinical validation approach in the face of lung Cancer. Biomark. Insights 19:11772719241287400. doi: 10.1177/11772719241287400, 39371614 PMC11452870

[ref73] Kubo S. Ninomiya R. Kajiwara T. Tokunaga A. Matsuda S. Murakami K. . (2025). *Helicobacter pylori* virulence factor CagA promotes snail-mediated epithelial-mesenchymal transition and invasive behavior by downregulating Semaphorin 5A in gastric epithelial cells. Biochem. Biophys. Res. Commun. 750:151421. doi: 10.1016/j.bbrc.2025.151421, 39892055

[ref74] Lange S. Ramirez M. I. (2021). Editorial: tissue remodeling in health and disease caused by Bacteria, parasites, Fungi, and viruses. Front. Cell. Infect. Microbiol. 11:642311. doi: 10.3389/fcimb.2021.642311, 33604310 PMC7884623

[ref75] Lee J. Kim H. Park J. S. (2024). Beyond the bile: exploring the microbiome and metabolites in cholangiocarcinoma. Life (Basel) 14. doi: 10.3390/life14060698, 38929681 PMC11204422

[ref76] Lee H. Lee H. K. Min S. K. Lee W. H. (2020). 16S rDNA microbiome composition pattern analysis as a diagnostic biomarker for biliary tract cancer. World J. Surg. Oncol. 18:19. doi: 10.1186/s12957-020-1793-3, 31980025 PMC6982396

[ref77] Li Z. Chu J. Su F. Ding X. Zhang Y. Dou L. . (2022). Characteristics of bile microbiota in cholelithiasis, perihilar cholangiocarcinoma, distal cholangiocarcinoma, and pancreatic cancer. Am. J. Transl. Res. 14, 2962–2971, 35702117 PMC9185071

[ref78] Li T. Coker O. O. Sun Y. Li S. Liu C. Lin Y. . (2025). Multi-cohort analysis reveals altered Archaea in colorectal Cancer fecal samples across populations. Gastroenterology 168, 525–538. doi: 10.1053/j.gastro.2024.10.023, 39490771

[ref79] Li M. Liu J. Zhu J. Wang H. Sun C. Gao N. L. . (2023). Performance of gut microbiome as an independent diagnostic tool for 20 diseases: cross-cohort validation of machine-learning classifiers. Gut Microbes 15:2205386. doi: 10.1080/19490976.2023.2205386, 37140125 PMC10161951

[ref80] Li Q. Xiao Y. Han L. Luo W. Dai W. Fang H. . (2025). Microbiome dysbiosis, neutrophil recruitment and mesenchymal transition of mesothelial cells promotes peritoneal metastasis of colorectal cancer. Nat. Cancer 6, 493–510. doi: 10.1038/s43018-025-00910-9, 39966610

[ref81] Li Y. Yu J. Zhang Y. Peng C. Song Y. Liu S. (2024). Advances in targeted therapy of cholangiocarcinoma. Ann. Med. 56:2310196. doi: 10.1080/07853890.2024.2310196, 38359439 PMC10877652

[ref82] Lin A. Jiang A. Huang L. Li Y. Zhang C. Zhu L. . (2025). From chaos to order: optimizing fecal microbiota transplantation for enhanced immune checkpoint inhibitors efficacy. Gut Microbes 17:2452277. doi: 10.1080/19490976.2025.2452277, 39826104 PMC12716052

[ref83] Liu Y. Hassan H. Brooks T. R. VanLith C. Cooley M. Elgozair M. . (2025). Blood and tissue dysregulated bile acids and short-chain fatty acids in cholangiocarcinoma. JHEP Rep. 7:101467. doi: 10.1016/j.jhepr.2025.101467, 41281446 PMC12637061

[ref84] Liu R. Li X. Hylemon P. B. Zhou H. (2018). Conjugated bile acids promote invasive growth of esophageal adenocarcinoma cells and Cancer stem cell expansion via sphingosine 1-phosphate receptor 2-mediated yes-associated protein activation. Am. J. Pathol. 188, 2042–2058. doi: 10.1016/j.ajpath.2018.05.015, 29963993 PMC6105923

[ref85] Liu J. Zhang Y. (2022). Intratumor microbiome in cancer progression: current developments, challenges and future trends. Biomark. Res. 10:37. doi: 10.1186/s40364-022-00381-5, 35642013 PMC9153132

[ref86] Liu Z. Zhang D. Chen S. (2024). Unveiling the gastric microbiota: implications for gastric carcinogenesis, immune responses, and clinical prospects. J. Exp. Clin. Cancer Res. 43:118. doi: 10.1186/s13046-024-03034-7, 38641815 PMC11027554

[ref87] Ma Y. Chen H. Li H. Zheng M. Zuo X. Wang W. . (2024). Intratumor microbiome-derived butyrate promotes lung cancer metastasis. Cell Rep. Med. 5:101488. doi: 10.1016/j.xcrm.2024.101488, 38565146 PMC11031379

[ref88] Ma Y. Chen T. Sun T. Dilimulati D. Xiao Y. (2024). The oncomicrobiome: new insights into microorganisms in cancer. Microb. Pathog. 197:107091. doi: 10.1016/j.micpath.2024.107091, 39481695

[ref89] Ma C. Han M. Heinrich B. Fu Q. Zhang Q. Sandhu M. . (2018). Gut microbiome-mediated bile acid metabolism regulates liver cancer via NKT cells. Science 360. doi: 10.1126/science.aan5931, 29798856 PMC6407885

[ref90] Ma J. Li J. Jin C. Yang J. Zheng C. Chen K. . (2023). Association of gut microbiome and primary liver cancer: A two-sample Mendelian randomization and case-control study. Liver Int. 43, 221–233. doi: 10.1111/liv.15466, 36300678

[ref91] Manivannan A. C. Dhandapani R. Velmurugan P. Thangavelu S. Paramasivam R. Ragunathan L. . (2022). Phage in cancer treatment - biology of therapeutic phage and screening of tumor targeting peptide. Expert Opin. Drug Deliv. 19, 873–882. doi: 10.1080/17425247.2022.2094363, 35748094

[ref92] Meacci D. Bruni A. Cocquio A. Dell'Anna G. Mandarino F. V. Marasco G. . (2025). Microbial landscapes of the gut-biliary Axis: implications for benign and malignant biliary tract diseases. Microorganisms 13. doi: 10.3390/microorganisms13091980, 41011314 PMC12472106

[ref93] Metwaly A. Kriaa A. Hassani Z. Carraturo F. Druart C. Arnauts K. . (2025). A consensus statement on establishing causality, therapeutic applications and the use of preclinical models in microbiome research. Nat. Rev. Gastroenterol. Hepatol. 22, 343–356. doi: 10.1038/s41575-025-01041-3, 40033063

[ref94] Mirzaei R. Afaghi A. Babakhani S. Sohrabi M. R. Hosseini-Fard S. R. Babolhavaeji K. . (2021). Role of microbiota-derived short-chain fatty acids in cancer development and prevention. Biomed. Pharmacother. 139:111619. doi: 10.1016/j.biopha.2021.111619, 33906079

[ref95] Miyabe K. Chandrasekhara V. Wongjarupong N. Chen J. Yang L. Johnson S. . (2022). Potential role of inflammation-promoting biliary microbiome in primary Sclerosing cholangitis and cholangiocarcinoma. Cancers (Basel) 14. doi: 10.3390/cancers14092120, 35565248 PMC9104786

[ref96] Nagahashi M. Yuza K. Hirose Y. Nakajima M. Ramanathan R. Hait N. C. . (2016). The roles of bile acids and sphingosine-1-phosphate signaling in the hepatobiliary diseases. J. Lipid Res. 57, 1636–1643. doi: 10.1194/jlr.R069286, 27459945 PMC5003161

[ref97] Nejman D. Livyatan I. Fuks G. Gavert N. Zwang Y. Geller L. T. . (2020). The human tumor microbiome is composed of tumor type-specific intracellular bacteria. Science 368, 973–980. doi: 10.1126/science.aay9189, 32467386 PMC7757858

[ref98] Park J. S. (2024). Microbiome and biliary tract Cancer. Korean J. Gastroenterol. 83, 1–5. doi: 10.4166/kjg.2023.135, 38268162 PMC12285506

[ref99] Patangia D. V. Anthony Ryan C. Dempsey E. Paul Ross R. Stanton C. (2022). Impact of antibiotics on the human microbiome and consequences for host health. Microbiology 11:e1260. doi: 10.1002/mbo3.1260, 35212478 PMC8756738

[ref100] Pérez P. N. Ramírez M. A. Fernández J. A. de Guevara L. L. (2014). A patient presenting with cholangitis due to Stenotrophomonas Maltophilia and *Pseudomonas aeruginosa* successfully treated with Intrabiliary Colistine. Infect. Dis. Rep. 6:5147. doi: 10.4081/idr.2014.5147, 25002957 PMC4083296

[ref101] Petrov G. Dymova M. Richter V. (2022). Bacteriophage-mediated Cancer gene therapy. Int. J. Mol. Sci. 23. doi: 10.3390/ijms232214245, 36430720 PMC9697857

[ref102] Phelps C. M. Willis N. B. Duan T. Lee A. H. Zhang Y. Rodriguez J. D. . (2025). Exercise-induced microbiota metabolite enhances CD8 T cell antitumor immunity promoting immunotherapy efficacy. Cell 188, 5680–5700. doi: 10.1016/j.cell.2025.06.018 40639377 PMC12258965

[ref103] Plieskatt J. L. Deenonpoe R. Mulvenna J. P. Krause L. Sripa B. Bethony J. M. . (2013). Infection with the carcinogenic liver fluke *Opisthorchis viverrini* modifies intestinal and biliary microbiome. FASEB J. 27, 4572–4584. doi: 10.1096/fj.13-232751, 23925654 PMC3804743

[ref104] Qi J. L. He J. R. Liu C. B. Jin S. M. Gao R. Y. Yang X. . (2020). Pulmonary *Staphylococcus aureus* infection regulates breast cancer cell metastasis via neutrophil extracellular traps (NETs) formation. MedComm 1, 188–201. doi: 10.1002/mco2.22, 34766117 PMC8491238

[ref105] Qian L. Wang L. Zou Z. Luan F. Cai X. Zhou J. . (2025). Distinct composition and metabolic potential of biliary microbiota in patients with malignant bile duct obstruction. Eur. J. Gastroenterol. Hepatol. 37, 585–593. doi: 10.1097/meg.0000000000002948, 40207470 PMC11949207

[ref106] Qu R. Zhang Y. Ma Y. Zhou X. Sun L. Jiang C. . (2023). Role of the gut microbiota and its metabolites in tumorigenesis or development of colorectal Cancer. Adv. Sci. (Weinh) 10:e2205563. doi: 10.1002/advs.202205563, 37263983 PMC10427379

[ref107] Qurashi M. Vithayathil M. Khan S. A. (2025). Epidemiology of cholangiocarcinoma. Eur. J. Surg. Oncol. 51:107064. doi: 10.1016/j.ejso.2023.107064, 37709624

[ref108] Rao B. Ren T. Wang X. Wang H. Zou Y. Sun Y. . (2021). Dysbiosis in the human microbiome of cholangiocarcinoma. Front. Physiol. 12:715536. doi: 10.3389/fphys.2021.715536, 34867436 PMC8633309

[ref109] Rao B. C. Zhang G. Z. Zou Y. W. Ren T. Ren H. Y. Liu C. . (2022). Alterations in the human oral microbiome in cholangiocarcinoma. Mil. Med. Res. 9:62. doi: 10.1186/s40779-022-00423-x, 36345047 PMC9641929

[ref110] Ridlon J. M. Alves J. M. Hylemon P. B. Bajaj J. S. (2013). Cirrhosis, bile acids and gut microbiota: unraveling a complex relationship. Gut Microbes 4, 382–387. doi: 10.4161/gmic.25723, 23851335 PMC3839982

[ref111] Saab M. Mestivier D. Sohrabi M. Rodriguez C. Khonsari M. R. Faraji A. . (2021). Characterization of biliary microbiota dysbiosis in extrahepatic cholangiocarcinoma. PLoS One 16:e0247798. doi: 10.1371/journal.pone.0247798, 33690612 PMC7943025

[ref112] Saha B. A T. R. Adhikary S. Banerjee A. Radhakrishnan A. K. Duttaroy A. K. . (2024). Exploring the relationship between diet, lifestyle and gut microbiome in colorectal cancer development: a recent update. Nutr. Cancer 76, 789–814. doi: 10.1080/01635581.2024.2367266, 39207359

[ref113] Salter S. J. Cox M. J. Turek E. M. Calus S. T. Cookson W. O. Moffatt M. F. . (2014). Reagent and laboratory contamination can critically impact sequence-based microbiome analyses. BMC Biol. 12:87. doi: 10.1186/s12915-014-0087-z, 25387460 PMC4228153

[ref114] Schöler D. Schnabl B. (2024). The role of the microbiome in liver disease. Curr. Opin. Gastroenterol. 40, 134–142. doi: 10.1097/mog.0000000000001013 38362864 PMC10990783

[ref115] Selway C. A. Eisenhofer R. Weyrich L. S. (2020). Microbiome applications for pathology: challenges of low microbial biomass samples during diagnostic testing. J. Pathol. Clin. Res. 6, 97–106. doi: 10.1002/cjp2.151, 31944633 PMC7164373

[ref116] Sender R. Fuchs S. Milo R. (2016). Revised estimates for the number of human and Bacteria cells in the body. PLoS Biol. 14:e1002533. doi: 10.1371/journal.pbio.1002533, 27541692 PMC4991899

[ref117] Sepich-Poore G. D. Zitvogel L. Straussman R. Hasty J. Wargo J. A. Knight R. (2021). The microbiome and human cancer. Science 371. doi: 10.1126/science.abc4552, 33766858 PMC8767999

[ref118] Shuwen H. Kefeng D. (2022). Intestinal phages interact with bacteria and are involved in human diseases. Gut Microbes 14:2113717. doi: 10.1080/19490976.2022.2113717, 36037202 PMC9427043

[ref119] Singh V. Yeoh B. S. Chassaing B. Xiao X. Saha P. Aguilera Olvera R. . (2018). Dysregulated microbial fermentation of soluble fiber induces cholestatic liver cancer. Cell 175, 679–694. doi: 10.1016/j.cell.2018.09.004 30340040 PMC6232850

[ref120] Sitthirak S. Suksawat M. Phetcharaburanin J. Wangwiwatsin A. Klanrit P. Namwat N. . (2022). Chemotherapeutic resistant cholangiocarcinoma displayed distinct intratumoral microbial composition and metabolic profiles. PeerJ 10:e13876. doi: 10.7717/peerj.13876, 35990899 PMC9390323

[ref121] Song J. Zhang W. Wang D. (2025). Gut microbiome in gastrointestinal neoplasms: from mechanisms to precision therapeutic strategies. Gut Pathog. 17:57. doi: 10.1186/s13099-025-00734-z, 40739580 PMC12309039

[ref122] Stewart O. A. Wu F. Chen Y. (2020). The role of gastric microbiota in gastric cancer. Gut Microbes 11, 1220–1230. doi: 10.1080/19490976.2020.1762520, 32449430 PMC7524314

[ref123] Sun M. Peng Z. Shen W. Guo X. Liao Y. Huang Y. . (2024). Synergism of *Fusobacterium periodonticum* and N-nitrosamines promote the formation of EMT subtypes in ESCC by modulating Wnt3a palmitoylation. Gut Microbes 16:2391521. doi: 10.1080/19490976.2024.2391521, 39193618 PMC11364064

[ref124] Sun J. Tang Q. Yu S. Xie M. Xie Y. Chen G. . (2020). Role of the oral microbiota in cancer evolution and progression. Cancer Med. 9, 6306–6321. doi: 10.1002/cam4.3206, 32638533 PMC7476822

[ref125] Takeuchi T. Nakanishi Y. Ohno H. (2024). Microbial metabolites and gut immunology. Annu. Rev. Immunol. 42, 153–178. doi: 10.1146/annurev-immunol-090222-102035, 38941602

[ref126] Tan C. C. S. Ko K. K. K. Chen H. Liu J. Loh M. Chia M. . (2023). No evidence for a common blood microbiome based on a population study of 9,770 healthy humans. Nat. Microbiol. 8, 973–985. doi: 10.1038/s41564-023-01350-w, 36997797 PMC10159858

[ref127] Tan J. H. Zhou W. Y. Zhou L. Cao R. C. Zhang G. W. (2020). Viral hepatitis B and C infections increase the risks of intrahepatic and extrahepatic cholangiocarcinoma: evidence from a systematic review and meta-analysis. Turk J Gastroenterol 31, 246–256. doi: 10.5152/tjg.2020.19056, 32343237 PMC7197930

[ref128] Tang Y. Xu L. Zhang G. Li K. Shi A. Shu L. . (2024). Survival analysis and prognostic nomogram for patients with cholangiocarcinoma after radical resection in Asia. Eur. J. Surg. Oncol. 50:108659. doi: 10.1016/j.ejso.2024.108659, 39243726

[ref129] Thanush D. Venkatesh M. P. (2023). Fecal microbiota transplantation: history, procedure and regulatory considerations. Presse Med. 52:104204. doi: 10.1016/j.lpm.2023.104204, 37944641

[ref130] Ting N. L. Lau H. C. Yu J. (2022). Cancer pharmacomicrobiomics: targeting microbiota to optimise cancer therapy outcomes. Gut 71, 1412–1425. doi: 10.1136/gutjnl-2021-326264, 35277453 PMC9185832

[ref131] Valle J. W. Lamarca A. Goyal L. Barriuso J. Zhu A. X. (2017). New horizons for precision medicine in biliary tract cancers. Cancer Discov. 7, 943–962. doi: 10.1158/2159-8290.Cd-17-0245, 28818953 PMC5586506

[ref132] Verdier J. Luedde T. Sellge G. (2015). Biliary mucosal barrier and microbiome. Viszeralmedizin 31, 156–161. doi: 10.1159/000431071, 26468308 PMC4569210

[ref133] Vieira-Silva S. Sabino J. Valles-Colomer M. Falony G. Kathagen G. Caenepeel C. . (2019). Quantitative microbiome profiling disentangles inflammation- and bile duct obstruction-associated microbiota alterations across PSC/IBD diagnoses. Nat. Microbiol. 4, 1826–1831. doi: 10.1038/s41564-019-0483-9, 31209308

[ref134] Wade K. H. Hall L. J. (2019). Improving causality in microbiome research: can human genetic epidemiology help? Wellcome Open Res. 4:199. doi: 10.12688/wellcomeopenres.15628.3, 32462081 PMC7217228

[ref135] Wang Z. Dan W. Zhang N. Fang J. Yang Y. (2023). Colorectal cancer and gut microbiota studies in China. Gut Microbes 15:2236364. doi: 10.1080/19490976.2023.2236364, 37482657 PMC10364665

[ref136] Wang X. Fang Y. Liang W. Wong C. C. Qin H. Gao Y. . (2024). *Fusobacterium nucleatum* facilitates anti-PD-1 therapy in microsatellite stable colorectal cancer. Cancer Cell 42, 1729–46.e8. doi: 10.1016/j.ccell.2024.08.019, 39303724

[ref137] Wang Y. Ma J. Cai W. Song M. Wang Z. Xu Z. . (2025). Fast encapsulation of microbes into dissolvable hydrogel beads enables high-throughput microbial single-cell RNA sequencing of clinical microbiome samples. Adv. Mater. 37:e2500481. doi: 10.1002/adma.202500481, 40200683

[ref138] Wang L. Qiao W. Zhen X. Zhang Y. Dong Z. (2025). Targeting the gut-liver axis in cholangiocarcinoma: mechanisms, therapeutic advances, and future directions. Front. Oncol. 15:1646897. doi: 10.3389/fonc.2025.1646897, 41018102 PMC12463604

[ref139] Wang Y. Wang Y. Zhou Y. Feng Y. Sun T. Xu J. (2024). Tumor-related fungi and crosstalk with gut fungi in the tumor microenvironment. Cancer Biol. Med. 21, 977–994. doi: 10.20892/j.issn.2095-3941.2024.0240, 39601429 PMC11667784

[ref140] Wang L. Zhao H. Wu F. Chen J. Xu H. Gong W. . (2025). Bile-liver phenotype: exploring the microbiota landscape in bile and intratumor of cholangiocarcinoma. Comput. Struct. Biotechnol. J. 27, 1173–1186. doi: 10.1016/j.csbj.2025.03.030, 40206347 PMC11981758

[ref141] Wekking D. Ende T. V. D. Bijlsma M. F. Vidal-Itriago A. Nieuwdorp M. Van Laarhoven H. W. M. (2025). Fecal microbiota transplantation to enhance cancer treatment outcomes across different cancer types: a systematic literature review. Cancer Treat. Rev. 140:103025. doi: 10.1016/j.ctrv.2025.103025, 41061376

[ref142] Wu N. Bayatpour S. Hylemon P. B. Aseem S. O. Brindley P. J. Zhou H. (2025). Gut microbiome and bile acid interactions: mechanistic implications for cholangiocarcinoma development, immune resistance, and therapy. Am. J. Pathol. 195, 397–408. doi: 10.1016/j.ajpath.2024.11.004, 39730075 PMC11841492

[ref143] Wu Y. Peng Y. (2024). Ten computational challenges in human virome studies. Virol. Sin. 39, 845–850. doi: 10.1016/j.virs.2024.04.008, 38697263 PMC11738758

[ref144] Xiao Y. Louwies T. Mars R. A. T. Kashyap P. C. (2024). The human microbiome-A physiologic perspective. Compr. Physiol. 14, 5491–5519. doi: 10.1002/cphy.c230013, 39109977 PMC12919717

[ref145] Xin H. Y. Zou J. X. Sun R. Q. Hu Z. Q. Chen Z. Luo C. B. . (2024). Characterization of tumor microbiome and associations with prognosis in intrahepatic cholangiocarcinoma. J. Gastroenterol. 59, 411–423. doi: 10.1007/s00535-024-02090-2, 38461467

[ref146] Yang Y. An Y. Dong Y. Chu Q. Wei J. Wang B. . (2024). Fecal microbiota transplantation: no longer cinderella in tumour immunotherapy. EBioMedicine 100:104967. doi: 10.1016/j.ebiom.2024.104967, 38241975 PMC10831174

[ref147] Yatsunenko T. Rey F. E. Manary M. J. Trehan I. Dominguez-Bello M. G. Contreras M. . (2012). Human gut microbiome viewed across age and geography. Nature 486, 222–227. doi: 10.1038/nature11053, 22699611 PMC3376388

[ref148] Ye C. Dong C. Lin Y. Shi H. Zhou W. (2023). Interplay between the human microbiome and biliary tract Cancer: implications for pathogenesis and therapy. Microorganisms 11. doi: 10.3390/microorganisms11102598, 37894256 PMC10608879

[ref149] Ye F. Shen H. Li Z. Meng F. Li L. Yang J. . (2016). Influence of the biliary system on biliary Bacteria revealed by bacterial communities of the human biliary and upper digestive tracts. PLoS One 11:e0150519. doi: 10.1371/journal.pone.0150519, 26930491 PMC4773253

[ref150] Ye S. H. Siddle K. J. Park D. J. Sabeti P. C. (2019). Benchmarking metagenomics tools for taxonomic classification. Cell 178, 779–794. doi: 10.1016/j.cell.2019.07.010, 31398336 PMC6716367

[ref151] Ye C. Zhang B. Lin Y. Han F. Shi H. Dong C. . (2025). Characteristics of gut microbiota and metabolites in extrahepatic cholangiocarcinoma and their prognostic value for resectable lesions. Front. Cell. Infect. Microbiol. 15:1523863. doi: 10.3389/fcimb.2025.1523863, 40028184 PMC11868125

[ref152] Yoon S. J. Han S. K. Kim T. S. Suk K. T. Choi D. H. Kim Y. D. . (2025). The crosstalk between gut microbiota and microbiota-derived metabolites in hepatocellular carcinoma. Crit. Rev. Microbiol. 51, 1315–1329. doi: 10.1080/1040841x.2025.2501590, 40468702

[ref153] Yoshimoto S. Loo T. M. Atarashi K. Kanda H. Sato S. Oyadomari S. . (2013). Obesity-induced gut microbial metabolite promotes liver cancer through senescence secretome. Nature 499, 97–101. doi: 10.1038/nature12347, 23803760

[ref154] Yu L. Hong Y. Maishi N. Matsuda A. Y. Hida Y. Hasebe A. . (2024). Oral bacterium *Streptococcus mutans* promotes tumor metastasis through thrombosis formation. Cancer Sci. 115, 648–659. doi: 10.1111/cas.16010, 38096871 PMC10859626

[ref155] Zeng R. Gou H. Lau H. C. H. Yu J. (2024). Stomach microbiota in gastric cancer development and clinical implications. Gut 73, 2062–2073. doi: 10.1136/gutjnl-2024-332815, 38886045 PMC11672014

[ref156] Zhang L. Chen C. Chai D. Kuang T. Deng W. Wang W. (2022). Alterations of gut mycobiota profiles in intrahepatic cholangiocarcinoma. Front. Microbiol. 13:1090392. doi: 10.3389/fmicb.2022.1090392, 36687597 PMC9853418

[ref157] Zhang Q. Ma C. Duan Y. Heinrich B. Rosato U. Diggs L. P. . (2021). Gut microbiome directs hepatocytes to recruit MDSCs and promote cholangiocarcinoma. Cancer Discov. 11, 1248–1267. doi: 10.1158/2159-8290.Cd-20-0304, 33323397 PMC8102309

[ref158] Zhang T. Zhang S. Jin C. Lin Z. Deng T. Xie X. . (2021). A predictive model based on the gut microbiota improves the diagnostic effect in patients with cholangiocarcinoma. Front. Cell. Infect. Microbiol. 11:751795. doi: 10.3389/fcimb.2021.751795, 34888258 PMC8650695

[ref159] Zhang Q. Zhou J. Zhai D. Jiang Q. Yang M. Zhou M. (2024). Gut microbiota regulates the ALK5/NOX1 axis by altering glutamine metabolism to inhibit ferroptosis of intrahepatic cholangiocarcinoma cells. Biochim. Biophys. Acta Mol. basis Dis. 1870:167152. doi: 10.1016/j.bbadis.2024.167152, 38582012

[ref160] Zhang N. Zhu W. Zhang S. Liu T. Gong L. Wang Z. . (2023). A novel *Bifidobacterium*/*Klebsiella* ratio in characterization analysis of the gut and bile microbiota of CCA patients. Microb. Ecol. 87:5. doi: 10.1007/s00248-023-02318-3, 38030815 PMC10687116

[ref161] Zhou C. B. Zhou Y. L. Fang J. Y. (2021). Gut microbiota in cancer immune response and immunotherapy. Trends Cancer 7, 647–660. doi: 10.1016/j.trecan.2021.01.010, 33674230

